# Duration of HIV-1 Viral Suppression on Cessation of Antiretroviral Therapy in Primary Infection Correlates with Time on Therapy

**DOI:** 10.1371/journal.pone.0078287

**Published:** 2013-10-25

**Authors:** Wolfgang Stöhr, Sarah Fidler, Myra McClure, Jonathan Weber, David Cooper, Gita Ramjee, Pontiano Kaleebu, Giuseppe Tambussi, Mauro Schechter, Abdel Babiker, Rodney E. Phillips, Kholoud Porter, John Frater

**Affiliations:** 1 Medical Research Council Clinical Trials Unit, London, United Kingdom; 2 Imperial College, London, United Kingdom; 3 The National Centre in HIV Epidemiology & Clinical Research and the University of New South Wales, Sydney, Australia; 4 Medical Research Council HIV Prevention Research Unit, Durban, South Africa; 5 Medical Research Council/Uganda Virus Research Institute Uganda Research Unit on AIDS, Entebbe, Uganda; 6 Vaccine and Immunotherapy Research Centre, San Raffaele Scientific Institute, Milan, Italy; 7 Universidade Federal do Rio de Janeiro, Rio de Janeiro, Brazil; 8 Nuffield Department of Clinical Medicine, Peter Medawar Building for Pathogen Research, Oxford, United Kingdom; 9 The Oxford Martin School, Peter Medawar Building for Pathogen Research, Oxford,United Kingdom; 10 Oxford National Institute for Health Research Biomedical Research Centre, Oxford, United Kingdom; Indiana University and Moi University, United States of America

## Abstract

**Objective:**

A minority of HIV-1 positive individuals treated with antiretroviral therapy (ART) in primary HIV-1 infection (PHI) maintain viral suppression on stopping. Whether this is related to ART duration has not been explored.

**Design:**

**And Methods**: Using SPARTAC trial data from individuals recruited within 6 months of seroconversion, we present an observational analysis investigating whether duration of ART was associated with post-treatment viraemic control. Kaplan-Meier estimates, logistic regression and Cox models were used.

**Results:**

165 participants reached plasma viral loads (VL) <400 copies/ml at the time of stopping therapy (ART stop). After ART stop, 159 experienced confirmed VL ≥400 copies/ml during median (IQR) follow-up of 167 (108,199) weeks.

Most participants experienced VL rebound within 12 weeks from ART stop, however, there was a suggestion of a higher probability of remaining <400 copies/ml for those on ART >12 weeks compared to ≤12 weeks (p=0.061). Cumulative probabilities of remaining <400 copies/ml at 12, 52 and 104 weeks after ART stop were 21% (95%CI=13,30), 4% (1,9), and 4% (1,9) for ≤12 weeks ART, and 32% (22,42), 14% (7,22), and 5% (2,11) for >12 weeks.

In multivariable regression, ART for >12 weeks was independently associated with a lower probability of being ≥400 copies/ml within 12 weeks of ART stop (OR=0.11 (95%CI=0.03,0.34), p<0.001)). In Cox models of time to VL ≥400 after 12 weeks, we only found an association with female sex (OR=0.2, p=0.001).

**Conclusion:**

Longer ART duration in PHI was associated with a higher probability of viral control after ART stop.

**Trial Registration:**

Controlled-Trials.com 76742797 http://www.controlled-trials.com/ISRCTN76742797.

## Introduction

Antiretroviral therapy (ART) suppresses viral replication to undetectable levels, but is unlikely to confer complete viral eradication [1,2]. Examples of HIV-1 positive individuals treated in primary HIV-1 infection (PHI) who maintain viral suppression when ART is stopped have received much attention as the mechanisms behind ‘post-treatment control’ (PTC) might inform strategies for achieving drug-free remission [3-5]. 

The timing and duration of therapy required to induce PTC is unclear, as are host and viral factors that may contribute. The SPARTAC (Short Pulse ART at Seroconversion) trial is the largest international randomised study of short-course therapy in PHI [6]. We present an analysis of the trial data investigating whether either 12 or 48 weeks of ART commenced within six months of the estimated date of seroconversion was associated with post-treatment viraemic control on cessation of therapy.

## Methods

### The SPARTAC trial

The protocol for the SPARTAC trial is available as supporting information; see Protocol S1. The supporting CONSORT checklist is published elsewhere [6]

### Ethics Statement

The SPARTAC trial was approved by the following authorities: Medicines and Healthcare products Regulatory Agency (UK), Ministry of Health (Brazil), Irish Medicines Board (Ireland), Medicines Control Council (South Africa), and The Uganda National Council for Science and Technology (Uganda). It was also approved by the following ethics committees in the participating countries: Central London Research Ethics Committee (UK), Hospital Universitário Clementino Fraga Filho Ethics in Research Committee (Brazil), Clinical Research and Ethics Committee of Hospital Clinic in the province of Barcelona, Spain, The Adelaide and Meath Hospital Research Ethics Committee (Ireland), University of Witwatersrand Human Research Ethics Committee, University of Kwazulu-Natal Research Ethics Committee and University of Cape Town Research Ethics Committee (South Africa), Uganda Virus Research Institute Science and ethics committee (Uganda), The Prince Charles Hospital Human Research Ethics Committee and St Vincent's Hospital Human Research Ethics Committee (Australia), and the National Institute for Infectious Diseases Lazzaro Spallanzani, Institute Hospital and the Medical Research Ethics Committee, and the ethical committee Of the Central Foundation of San Raffaele, MonteTabor (Italy). All participants signed a written informed consent.

### Patients and statistical analyses

366 adults were randomized within six months of the estimated date of HIV-1 seroconversion to receive ART for 48 weeks (ART-48, n=123), 12 weeks (ART 12, n=120) or none (Standard Care, n=123). The criteria for diagnosing PHI are described elsewhere [6]. 

For participants randomised to either the ART 12 or ART 48 trial arms, we included data from those with both an HIV-1 RNA measurement available within 14 days prior to stopping ART and at least one subsequently, and who had plasma viral loads <400 copies/ml at the time of stopping. We estimated time to viral rebound (≥400 copies/ml) using time-to-event methods, censoring at time last seen or on starting long-term ART. We assessed whether the probability of maintaining an undetectable viral load <400 copies/ml following ART stop was associated with ART duration using a log-rank test and a 12-week cut-off to differentiate between two ART groups. Analyses were undertaken using actual duration of therapy rather than according to trial randomisation.

To explore virological control in participants who did not receive ART in PHI, we analysed data from those who never commenced therapy and with documented HIV-1 plasma viral load measurements available at recruitment and then subsequently.

‘Log-log’ plots (data not shown) showed that the hazards in the two ART groups (≤12 or >12 weeks) were not proportional over the whole analysis time, so we used logistic regression to first examine the effect of the duration of therapy on HIV-1 RNA rebound by 12 weeks after stopping ART. We adjusted for sex, site (African vs non-African), age, baseline RNA, baseline CD4, and interval between estimated seroconversion and ART initiation. We then used Cox proportional hazard models to examine this effect on time to RNA ≥400 copies/ml after 12 weeks from ART stop for those remaining undetectable. We repeated the analyses restricted to data from the subset of individuals at (non-African) sites where HIV-1 RNA assays with detection limits < 50 copies/ml were used.

## Results

### Patient Selection and Demographics

Of 243 participants in SPARTAC randomised to receive ART, 5 did not start ART, and 5 started but did not stop ART. Of the 233 remaining, 39 had no HIV-1 RNA measurement available at the time of stopping ART. Of the 194 with available plasma HIV-1 RNA data, 165 (85%) reached <400 copies/ml at the time of ART cessation (86 and 79, for ART 12 and ART 48, respectively). The actual duration of ART received was a median (IQR, range) of 12 (12 to 12, 11 to 16) and 48 (48 to 48, 12 to 53) weeks for ART 12 and ART 48, respectively. The majority (n=110) were male, most of whom were infected through sex between men (n=101). At randomisation, the median (IQR) age was 34 (27 to 41) years, the estimated time since seroconversion was 85 (IQR, 60 to 101) days, the CD4 cell count was 565 (IQR, 463 to 707) cells/mm^3^ and the log_10_ HIV-1 RNA was 4.39 (IQR, 3.66 to 5.04) copies/ml of plasma (Table 1). Fourteen (8%) of the 165 participants had HIV-1 RNA ≤400 copies/ml at randomisation, before ART initiation.

**Table 1:** Baseline characteristics for participants in SPARTAC analysed in this study.

**Baseline variable**		**Participants who did not start ART**	Participants who started and stopped ART*	**Overall study population**	**Study population: ART ≤12 weeks**	**Study population: ART >12 weeks**
		(n=127)	(n=233)	(n=165)	(n=86)	(n=79)
Randomised arm:	no ART	122 (96%) ^♯^	0	0	0	0
	ART 12	0	120 (52%)	86 (52%)	85 (99%)	1 (1%)
	ART 48	5 (4%)	113 (49%)	79 (48%)	1 (1%)	78 (99%)
Sex:	Male	74 (58%)	139 (60%)	110 (67%)	52 (60%)	58 (73%)
	Female	53 (42%)	94 (40%)	55 (33%)	34 (40%)	21 (27%)
Site:	Africa	49 (39%)	88 (38%)	49 (30%)	31 (36%)	18 (23%)
	Other	78 (61%)	145 (62%)	116 (70%)	55 (64%)	61 (77%)
Risk group:	MSM^+^	72 (57%)	127 (55%)	101 (61%)	48 (56%)	53 (67%)
	Heterosexual	54 (43%)	104 (45%)	62 (38%)	37 (43%)	25 (32%)
	Other / unknown	1 (1%)	2 (1%)	2 (1%)	1 (1%)	1 (1%)
Age	(years)	31 (25-38)	32 (24-40)	34 (27-41)	33 (26-40)	34 (28-43)
Time from estimated seroconversion to randomisation	(days)	79 (58-103)	85 (63-106)	85 (60-101)	82 (63-100)	86 (57-102)
CD4	(cells/mm^3^)	557 (413-724)	560 (445-700)	565 (463-707)	543 (464-670)	600 (455-735)
Log_10_ HIV-1-RNA	(copies/mL)	4.68 (3.68-5.24)	4.42 (3.66-5.13)	4.39 (3.66-5.04)	4.24 (3.49-4.68)	4.66 (3.96-5.24)

Values are number (%) or, for continuous variables, median (interquartile range).

* Includes patients randomised to either the ART 12 and ART 48 arms. Of the 243 participants recruited to receive ART, 5 never started (and are included in the untreated analysis detailed in the first column) and 5 started but never stopped, and have been excluded from the analysis.

♯ Of 123 participants in the Standard Care arm, 1 did not have baseline RNA measurements available and was excluded.

+ Men who have sex with men.

### Analysis of ‘post treatment control’ in SPARTAC

On stopping ART, 159 of the 165 participants experienced a confirmed viral load rebound ≥400 RNA copies/ml (i.e. on two consecutive occasions) during a median (IQR) follow-up of 167 (108 to 199) weeks, censored at the time of commencing long-term ART. A further two participants had one HIV-1 RNA measurement ≥400 copies/ml within one month of stopping ART, followed by initiation of long-term ART or loss to follow-up. The remaining four participants (1 ART 12, 3 ART 48) remained <400 copies/ml for 164-202 weeks, three of whom experienced one to four HIV-1 RNA blips ≥400 copies/ml. The pre-therapy baseline viral loads for these 4 individuals were: 13,000 and 18,600 copies/ml for one, 1090 and 459 copies/ml for the second, <400 copies/ml for the third, and <100 copies/ml for the fourth (ART 12 recipient). The CD4 cell counts on stopping ART for these participants were between 769-1429 cells/mm^3^. 

### Analysis of time to plasma viral load rebound

The majority of participants experienced an HIV-1 plasma RNA rebound within 12 weeks from stopping ART, with overall Kaplan-Meier estimates for the probability of remaining <400 copies/ml at 12, 52 and 104 weeks of 26% (95% CI, 20 to 33), 9% (95% CI, 5 to 14) and 4% (95% CI, 2 to 8), respectively.

**Figure 1:**
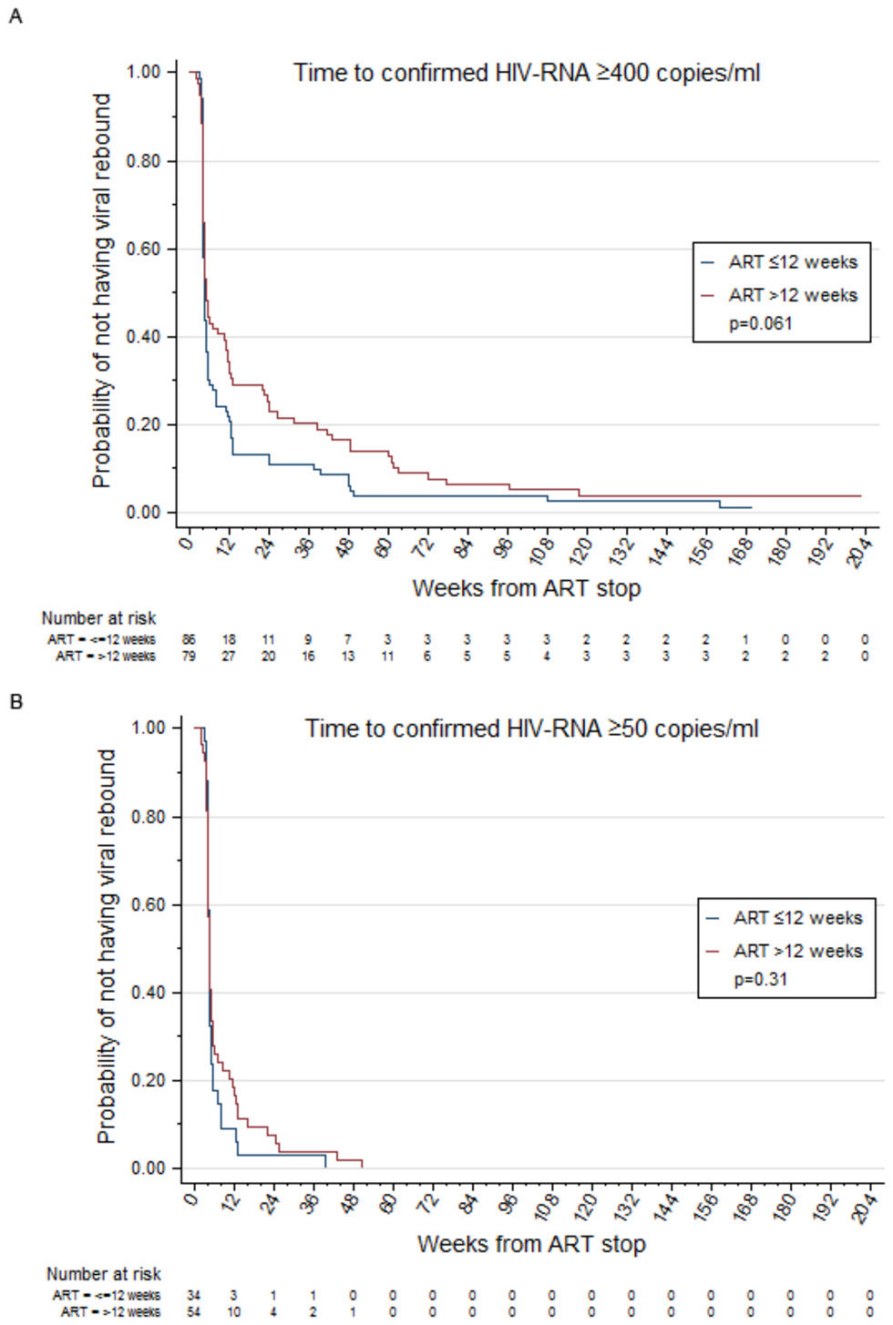
Time to confirmed plasma HIV-1 RNA detection by duration of ART. Kaplan Meier survival analysis showing time to virological rebound defined as (a.) <400 HIV-1 RNA copies/ml of plasma and, (b.) <50 RNA copies/ml of plasma. The x-axis shows weeks since cessation of ART. The y-axis shows proportions of participants remaining undetectable. Numbers at risk and contributing to the analysis at each time-point are shown below the x-axis. ART: antiretroviral therapy; VL = viral load.

Comparing participants who received >12 weeks of ART compared with ≤12 weeks revealed a statistically non-significant higher probability of remaining <400 copies/ml after stopping therapy for those on ART for >12 weeks (p=0.061). Cumulative probabilities of remaining <400 copies/ml at 12, 52 and 104 weeks after stopping ART were estimated to be 32% (95% CI, 22 to 42), 14% (95% CI, 7 to 22), and 5% (95% CI, 2 to 11) for those prescribed >12 weeks ART, compared with 21% (95% CI, 13 to 30), 4% (95% CI, 1 to 9), and 4% (95% CI, 1 to 9) for those receiving ≤12 weeks ART (Figure 1a).

When exploring the probability of viral load rebound within the first 12 weeks after stopping therapy, we found strong evidence that taking ART for >12 weeks was independently associated with maintaining <400 copies/ml (Odds Ratio 0.11 (95% CI, 0.03 to 0.34), p<0.001), as was female sex (Odds Ratio 0.28 compared to men (95% CI, 0.10 to 0.80), p=0.017). Higher baseline HIV-1 viral load prior to starting ART was associated with a higher probability of viral rebound to ≥400 RNA copies/ml (4.08 per one log_10_ increase (2.32 to 7.17), p<0.001). There was no evidence that having HIV-1 RNA ≥400 copies/ml after stopping ART was associated with the interval between estimated time since seroconversion and ART initiation (p=0.25), age (p=0.74), or CD4 cell count at randomisation (p=0.36). 

In univariable and multivariable (all covariates as in previous models except for sex) analyses, participants from African sites also had a lower probability of HIV-1 RNA ≥400 copies/ml during the first 12 weeks after stopping ART, however, as all African participants were female this effect could not be disentangled from that of sex. In sensitivity analyses, the effect of ART duration remained practically unchanged when African participants were excluded (OR 0.08 (95%CI, 0.01 to 0.44), p=0.004) or those with HIV-1 RNA <400 copies/ml at randomisation (i.e. before start of ART), or whether we included a factor in the model for having HIV-1 RNA <400 copies/ml at randomisation (data not shown). 

In Cox models exploring determinants for viral rebound more than 12 weeks after stopping therapy (in those who were <400 copies/ml at 12 weeks), we only found an association with sex (Hazard Ratio 0.2 compared to men (95%CI, 0.1 to 0.5), p=0.001), but not with duration of ART, baseline HIV-1 RNA or any other factor.

Restricting analyses to the subset of participants (n=126) recruited at centres using an HIV-1 RNA assay with a detection threshold of 50 copies/ml, 88 (70%) achieved a plasma HIV-1 RNA <50 copies/ml. One participant had HIV-1 RNA <50 copies/ml at randomisation. For these 88 participants (Figure 1b), HIV-1 RNA rebound occurred much sooner, and only 14% of participants (95% CI, 7 to 22) remained <50 copies/ml at 12 weeks, and none beyond 52 weeks after ART stop. Cumulative probabilities of remaining <50 copies/ml at 12 weeks after ART stop were 9% (95% CI, 2 to 21) and 17% (95% CI, 8 to 28) for those treated for ≤12 weeks and >12 weeks, respectively (p=0.31) (Figure 1b). Multivariable logistic regression of having confirmed HIV-1 RNA ≥50 copies/ml during the first 12 weeks from ART stop showed trends similar to the analysis using the 400 copies/ml threshold for the effect of ART duration (p=0.13) and HIV-1 RNA at randomisation (p=0.08).

Of the 127 who did not initiate ART and had HIV-1 RNA measurements available at baseline and subsequently (122 randomised to no therapy and 5 who had been, but did not initiate it), 11 had plasma viral loads <400 copies/ml at randomisation, one of whom did not experience confirmed HIV-1 RNA rebound during nearly 4 years of follow-up, although experiencing 2 blips in the first year. The remaining 10 participants all experienced confirmed HIV-1 RNA ≥ 400 copies/ml within the 1^st^ (n=5), 2^nd^ (n=1), 3^rd^ (n=1), and 4^th^ (n=3) year of follow-up, respectively (median 96 weeks). Three participants who did not initiate ART and had detectable viraemia (HIV-1 RNA ≥400 copies/ml) at baseline achieved a viral load <400 copies/ml within 12 months; two rebounded after 4 and 99 weeks, and the other remained <400 copies for 233 weeks.

## Discussion

We analysed data from participants enrolled in SPARTAC sampled out to a median of 167 weeks, showing that the majority (98%) had rebound viraemia on cessation of ART - only four participants maintained HIV-1 RNA <400 copies/ml on stopping therapy. Given their low pre-therapy baseline viral loads, it is possible that they may have controlled spontaneously, making it difficult to distinguish between the two phenotypes (i.e. spontaneous control vs PTC) [7]. This would be consistent with the finding that 3% of untreated participants in SPARTAC spontaneously controlled viral replication in the first 3 years following seroconversion (4 of 127). Other studies have also reported spontaneously controlled viraemia in 3.7-6.7% of untreated individuals [7-10].

We did not identify individuals with prolonged viral suppression after therapy equivalent in number to those described elsewhere [3,11]. However, we find evidence for an independent association between the duration of treatment in PHI and the likelihood of viral load rebound. Our data suggest that spontaneous treatment-independent suppression alone cannot explain these findings, which is supported by recent findings from the VISCONTI study which suggest that PTCs and spontaneous controllers are likely to be two separate phenotypes [11]. Although other cohorts have reported results suggestive of PTC [12], none of these studies found evidence of a treatment duration effect on the probability of PTC. 

Our data from SPARTAC are consistent with a transient effect of ART in PHI on viral suppression on stopping therapy. 14% of participants were <400 copies/ml at 52 weeks if they had received 12 or more weeks of ART in PHI, a higher proportion than those receiving less than 12 weeks. Our study is limited by the fact that the maximum treatment duration before stopping was 53 weeks. It may, therefore, be possible to achieve higher proportions (or even sustained control) with a longer ART duration. 

The impact of therapy at PHI on subsequent viraemia and clinical progression remains a clinically and mechanistically important question. SPARTAC demonstrated clinical benefit and suppression of viraemia with 48 (but not 12) weeks of short-course ART, and the analyses presented here demonstrate that time on therapy is associated with the duration of viral suppression on stopping. With a median follow up of 167 weeks, we find no convincing evidence from our dataset of sustained PTC. Further comparison of patients from different cohorts would benefit from agreed criteria against which to test PTC, which would assist with discrimination from spontaneous control. Data from the VISCONTI cohort suggest that the mechanism of PTC is distinct from elite controllers in that there is no enrichment for favourable HLA Class I alleles and no evidence of strong T cell immunity [11]. The proviral reservoir is low in both cases, and whether this is mechanistic demands further investigation in larger studies. 

Three factors in our study population were strongly correlated with viraemic control: African site, sex and HIV RNA threshold used (i.e. <50 versus <400 copies/ml). Of these parameters it is not possible to discriminate between effects: both female sex and African site were strong predictors of remaining < 400 copies/ml for longer, however, all participants from African sites were female, and there were only 6 women from non-African sites.

The impact of sex has been reported previously. In the CASCADE cohort, amongst untreated individuals with PHI, women were more likely to achieve undetectable viraemia [10]. In the ANRS CO6 PRIMO study, women recruited at PHI were more likely to achieve post-treatment control after ART interruption compared with men [5]. Our findings are therefore consistent with these other reports, however due to our study design we cannot disentangle sex from other possible factors. These data warrant further investigation, and consideration of sex needs to be incorporated into future study designs, to allow an independent effect to be determined and underlying mechanisms explored.

Studies on the impact of site (African vs non-African), or associated HIV-1 subtypes, can be confounded by socio-economic or epidemiological factors, and the published evidence for an impact on clinical progression is not strong [13-15]. It is not surprising that using data from participants with suppression below 50 HIV-1 RNA copies/ml was associated with faster rebound compared with 400 RNA copies/ml. The assay with a detection threshold of 400 copies/ml was only used in the African sites, and therefore exclusion of these sites removes participants with lower risk of rebound (most females and all African participants). It is not possible to infer in this study whether the choice of assay impacted the observed times to rebound, but the evidence for an enhanced ‘post-treatment control’ effect in the African participants warrants further investigation.

In conclusion, we find a transient post-treatment effect on viral suppression, which supports the arguments for initiating ART in PHI. Further studies to understand the mechanisms behind this effect will help determine whether long-term virological remission is an achievable goal.

## Supporting Information

Protocol S1 **Trial Protocol.**
(PDF)
