# Supplementary material for: Duration of HIV-1 Viral Suppression on Cessation of Antiretroviral Therapy in Primary Infection Correlates with Time on Therapy
Source: PLoS One. 2013 Oct 25;8(10):e78287. doi: 10.1371/journal.pone.0078287 (PMC3808338; doi:10.1371/journal.pone.0078287)
Supplement: Protocol S1 — Trial Protocol. (PDF) [file pone.0078287.s001.pdf]

Short Pulse AntiRetroviral Therapy At HIV seroConversion

A Multi-Centre Randomised Trial of Therapeutic Intervention at Primary HIV-1 Infection

Protocol Number 6.0

Protocol Date 20.04.09

Trial Sponsor
Imperial College
Exhibition Road
London SW7 2AZ

Funded by
Wellcome Trust
183 Euston Road
London NW1 2BE

Chief Investigator

Prof Jonathan Weber
Imperial College
Wright-Fleming Institute, Norfolk Place,
London W2 1PG, UK
j.weber@imperial.ac.uk
Tel: +44 20 7594 3905
Fax: +44 20 7594 3906

Principal Investigators

Prof David Cooper
Level 4
Lowy Packer Building
St Vincent's research Precinct
405 Liverpool St
Darlinghurst, NSW, 2010
Australia
dcooper@nchecr.unsw.edu.au
Tel: +61 83824904
Fax: +61 2 9385 0920

Dr Pontiano Kaleebu
MRC Unit on AIDS in Uganda
Uganda Virus Research Institute
P.O Box 49, Entebbe
Uganda
pontiano.kaleebu@mrcuganda.org
Tel: +256 41 320272/320042
Fax: +256 41 321137

Dr Gita Ramjee
HIV Prevention Research Unit
Medical Research Council
PO Box 70380
Overport
4067
Durban, Kwazulu-Natal
ramjeeg@mrc.ac.za
Tel: +27 (31) 202 0777 ext 223
Fax: +27 (31) 202 0950

Dr Helen Rees
Reproductive Health and HIV Research Unit
University of the Witwatersrand
P.O Box 181512, Hillbrow, 2038
Johannesburg, South Africa
hrees@rhru.co.za
Tel: +2711 3584501
Fax: +27 11 3585301

Dr Mauro Schechter
Head, AIDS Research Laboratory,
Hospital Universitario Clementino Fraga Filho,
Universidade Federal do Rio de Janeiro,
Av. Brigadeiro Trompowski s/n – Ilha do Fundao,
Rio de Janeiro – RJ – Brazil.
CEP: 21.941-590
maurosch@hucff.ufrj.br
Tel: +55 21 2562 2725
Fax: +55 21 2590 1615

	Dr Giuseppe Tambussi<br>Department of Infectious Diseases<br>San Raffaele Hospital<br>Via Stamira d'Ancona, 20<br>20127 Milan<br>Italy	g.tambussi@hsr.it<br>Tel: +39 02 2643 7960<br>Fax: +39 02 2643 7903
	Prof Robin Wood<br>Desmond Tutu HIV Centre<br>P O Box 13801<br>Mowbray<br>7705	robinwood@hiv-research.org.za<br>Tel: +27 21 650 6955<br>Fax: +27 21 650 6963
	Dr Jose Maria Miro Meda<br>Hospital Clinic<br>Barcelona<br>Spain	jmmiro@ub.edu<br>Tel: +34-93-2275400<br>Fax: +34-93-4514438
**Trial physicians**	Dr Sarah Fidler<br>Imperial College<br>St. Mary's Hospital<br>London W2 1NY, UK	s.fidler@imperial.ac.uk<br>Tel: +44 20 7594 3903<br>Fax: +44 20 7594 3906
	Prof Tony Kelleher<br>Level 9<br>Lowy Packer Building<br>St Vincent's research Precinct<br>405 Liverpool St<br>Darlinghurst, NSW, 2010<br>Australia	t.kelleher@cfi.unsw.edu.au<br>Tel: +61 2 8382 2094<br>Fax: +61 8382 4967
	Dr Paula Gama<br>Clinical Trials Unit - Projeto Praça Onze,<br>Hospital Escola São Francisco de Assis,<br>Universidade Federal do Rio de Janeiro,<br>Av. Presidente Vargas 2863 – 3º Andar,<br>Centro - Rio de Janeiro - RJ - Brazil.<br>CEP: 20.210-030	gama@ponze.ufrj.br<br>Tel: +55 21 2273 9073<br>Fax: +55 21 2504-2031
**Trial immunologists**	Prof Rodney Phillips<br>(PA- Suneeta Garewal)<br>Peter Medawar Centre<br>South Parks Road<br>Oxford OX1 3SY, UK	Rodney.Phillips@ndm.ox.ac.uk<br>Tel: +44 1865 281 230<br>Fax: +44 1865 281 890

**Trial virologists**	Dr Phillip Cunningham<br>Level 4<br>Lowy Packer Building<br>St Vincent's research Precinct<br>405 Liverpool St<br>Darlinghurst, NSW, 2010<br>Australia	p.cunningham@cfi.unsw.edu.au<br>Tel: +61 2 8382 2388<br>Fax: +61 2 8382 2391
	Dr Pontiano Kaleebu<br>MRC Unit on AIDS in Uganda<br>Uganda Virus Research Institute<br>P.O Box 49, Entebbe<br>Uganda	pontiano.kaleebu@mrcuganda.org<br>Tel: +256 41 320272/320042<br>Fax: +256 41 321137
	Prof Myra McClure<br>Imperial College<br>Wright-Fleming Institute<br>London W2 1NY, UK	m.mcclure@imperial.ac.uk<br>Tel: +44 20 7594 3902<br>Fax: +44 20 7594 3906
	Wendy Stevens<br>Contract Lab Services (CLS)<br>Room 29, Area 454,<br>Johannesburg Hospital<br>Parktown<br>Johannesburg 2193<br>South Africa	wendy.stevens@nhls.ac.za<br>Tel: +27 11 489 8505<br>Fax: +27 11 484 5812
**Trial Statistician**	Prof Abdel Babiker<br>MRC CTU<br>222 Euston Road<br>London NW1 2DA. UK	a.babiker@ctu.mrc.ac.uk<br>Tel: +44 20 7670 4708<br>Fax: +44 20 7670 4818
**Trial Management & Co-ordination**	Michelle Gabriel<br>MRC CTU<br>222 Euston Road<br>London NW1 2DA. UK	mg@ctu.mrc.ac.uk<br>Tel: +44 20 7670 4813<br>Fax: +44 20 7670 4814
	Dr Kholoud Porter<br>MRC CTU<br>222 Euston Road<br>London NW1 2DA. UK	k.porter@ctu.mrc.ac.uk<br>Tel: +44 20 7670 4715<br>Fax: +44 20 7670 4815
	Dr Katherine Boyd<br>MRC CTU<br>222 Euston Road<br>London NW1 2DA. UK	k.boyd@ctu.mrc.ac.uk<br>Tel: +44 20 7670 4730<br>Fax: +44 20 7670 4814

Compliance

The study will be conducted in compliance with the protocol, MRC GCP, Data Protection Act (DPA number g0027154) and ICH

CONTENTS

1 AMENDMENTS 7
2 SUMMARY 10
2.1 Abstract 10
3 BACKGROUND 12
3.1 Introduction 12
3.2 Justification for the trial 13
3.3 Pilot study 13
3.4 Choice of drugs 14
4 GENERAL PLAN 15
4.1 Objectives 15
4.2 Design 15
4.3 Efficacy measures 16
4.4 Toxicity measures 17
4.5 Adherence measure 17
4.6 Eligibility and exclusions 17
4.7 Concomitant medication 18
4.8 Substudies 18
4.9 Subanalyses 18
4.10 Co-enrolment of Spartac Patients on Other Studies 19
5 PROCEDURES 22
5.1 Screening 22
5.2 Trial entry 22
5.3 Randomisation 23
5.4 Routine follow-up 23
5.5 Procedures for assessing efficacy 24
5.6 Schedule of visits and investigations 25
6 VIROLOGICAL AND IMMUNOLOGICAL SPECIMENS AND STUDIES 26
6.1 Virology 26
6.2 Immunology 27
7 ADVERSE EVENTS 29
7.1 Definition 29
7.2 Grading of adverse events 29
7.3 Severe grade adverse events 29
7.4 Serious grade adverse events 29
7.5 Reporting grades 3 & 4 and serious adverse events 30
8 WITHDRAWAL BY SUBJECTS 30
9 ANTIRETROVIRAL MEDICATION 31
9.1 Dispensing 31
9.2 Modification of trial treatment 31
9.3 Initiation of late treatment 31
9.4 Hepatitis co-infection and use of short courses of ART 31
10 STATISTICS 32
10.1 Method of randomisation 32
10.2 Sample size 32
10.3 Monitoring and interim analysis 32
10.4 Analysis plan 33
11 ETHICAL CONSIDERATIONS 33
11.1 Subject payment 33
12 INDEMNITY 33

13 TRIAL COMMITTEES **33**
13.1 Trial management group 33
13.2 Trial steering group 33
13.3 Data Monitoring And Ethics Committee 34
14 PUBLICATIONS **34**
15 REFERENCES **35**

APPENDICES **39**
APPENDIX 1: ANTIRETROVIRAL DRUG INFORMATION 39
APPENDIX 2: PATIENT INFORMATION SHEET AND CONSENT FORMS 42
APPENDIX 3: GP LETTER 48
APPENDIX 4: DEFINITIONS AND CRITERIA FOR AIDS EVENTS 49
APPENDIX 5: ADHERENCE QUESTIONNAIRE 52
APPENDIX 6: GRADING OF ADVERSE EVENTS 53
APPENDIX 7: TRIAL CO-INVESTIGATORS AND COORDINATING CENTRES 55
APPENDIX 8: LONG-TERM STORAGE OF SAMPLES PATIENT INFORMATION SHEET AND CONSENT FORMS 58

Glossary and Abbreviations

ALT	Alanine aminotransferase
ABPI	Association of the British Pharmaceutical Industry
ART	Antiretroviral therapy
AST	Aspartate aminotransferase
CRF	Case record form
CTU	Clinical Clinical Trials Unit
DSMC	Data and Safety Monitoring Committee
GCP	Good Clinical Practice
GP	General Practitioner
HAART	Highly Active Antiretroviral Therapy
HIV	Human Immunodeficiency Virus
IAS	International AIDS Society
ICH	International Conference on Harmonisation
LCART	Long course anti-HIV therapy
LTX	Late anti-HIV treatment which may be administered according to local HIV treatment guidelines when indicated
MRC	Medical Research Council
NCHECR	National Centre in HIV Epidemiology and Clinical Research
NRTI	Nucleoside Reverse Transcriptase Inhibitor
NNRTI	Non-Nucleoside Reverse Transcriptase Inhibitor
PBMC	Peripheral blood monocytes
PCR	Polymerase chain reaction
PHI	Primary HIV Infection
PI	Protease Inhibitor
PTX	Primary anti-HIV treatment given to patients on arms A & B
QA	Quality Assurance
RT	Reverse transcriptase
SAE	Serious adverse event
SCART	short course anti-HIV therapy
TB	Tuberculosis
TMG	Trial Management Group
TSC	Trial Steering Committee
ULN	Upper limit of normal

1 AMENDMENTS

Amendments to Protocol number 1.0 dated 19/07/04 (approved by MREC London as version 0.9.4, approved by AMNCH REC Dublin as version 0.9.4, approved by St.Vincent's Hospital Sydney HREC as version 0.1, approved by Uni. Of Witwatersrand Johannesburg as version 1.0)

1. Revised final protocol (number 1.1 date 29/04/05) with amendments to number 1.0

- **Contacts page.** Removed Sharon Cassol's laboratory and replaced with Wendy Steven's laboratory. Replaced Dr John Clarke with Prof Myra McClure. Removed Dr Don Smith.
- **Section 2.1, 5.3.1, 6.2.1.2, 9.1.2, contacts page.** Removed references to participation of Russian sites.
- **Section 4.2.2.**
  Added "(this is the recommended regimen)" after protease inhibitor in the sentence "The PTX regimen chosen should contain at least two nucleoside reverse transcriptase inhibitors with either a protease inhibitor or a non-nucleoside reverse transcriptase inhibitor".

  Deleted the paragraph "Due to delay in receiving resistance test results from randomisation (baseline) of up to four weeks it will not be possible to select PTX based on this information. When the patient returns for visit 3 (week 4) the result will be available and any changes needed can be made at this visit."

  Added the paragraph "A genotypic resistance test will be requested on all patients at the screening visit to allow the results to be available to the clinician before randomisation so that PTX may be selected based on this information."

  The sentence "Upon stopping PTX for arms A and B, where an NNRTI has been used as part of the regimen, the other antiretrovirals will need to be continued alone for 5 days after stopping the NNRTI" replaced with "Upon stopping PTX for arms A and B, where an NNRTI has been used as part of the regimen, it is recommended that the NNRTI should be stopped at week 12 (arm A) or week 48 (arm B), but the other antiretrovirals need to be continued alone for 2 weeks. However, this time period should only be used for guidance, as the current lack of data does not allow for a definite optimum staggered stop for NNRTI used in HAART regimens."
- **Section 4.3.2.** "Slope of CD4 decline" and "Differences in blood pressure from randomisation at week 12 and week 48" added to list of secondary outcomes.
- **Section 4.6.** New section added on blood pressure sub-analysis.
- **Section 5.1.** "Genotypic resistance test" added to list of screening tests
- **Section 5.2.**
  Deleted the paragraph "Patients with the following measurements above the 'upper limit of normal' (ULN) are excluded from trial entry: Creatinine >ULN, Amylase, Lipase >1.5 times ULN (entry allowed, however, if pancreatic amylase is <1.5 times ULN), patients with haemoglobin <9.5g/dl; neutrophils <0.75 10^9/l; or platelets <50 10^9/l are also excluded."

  Added "for opportunistic infections and anti-hypertension" to the end of the sentence "a) recording the clinical history since screening, including AIDS events, presence of non-HIV related adverse events, and current use of concomitant medication"
- **Section 5.4.**
  Added "and anti-hypertension" after opportunistic infections in the sentence "a) Clinical history since last assessment, including the time of the last doses of trial drugs taken, AIDS events, presence of non-HIV related adverse events, use of concomitant medication for opportunistic infections, and occurrence of pregnancy."
- **Section 5.6.**
  "Genotypic resistance test" done at Visit 2 Randomisation moved to Visit 1 Screening.

  In the key of the chart, "arm A if HIV RNA is >50 copies/ml" replaced with "arm A if HIV RNA detectable"
- **Section 9.2.**
  The paragraph "LCART arm at visit 6 (week 24) if plasma viral load is not less than 50 copies/ml an additional sample must be taken for genotyping. If evidence of drug resistance has developed since baseline the PTX regime must be changed within 4 weeks of result and patient will be asked to attend for an additional visit. Adherence must be addressed at this point" replaced with "LCART arm at visit 6 (week 24) if plasma viral load is detectable a genotypic resistance test should be attempted on an additional sample taken at that visit. If evidence of drug resistance has developed since screening the

PTX regime must be changed within 4 weeks of result and patient will be asked to attend for an additional visit. Adherence must be addressed at this point".

2. Revised protocol (number 2.0 date 31/03/06) with amendments to number 1.1 date 29/04/05

• **Contacts page.** Added Principal Investigators for Uganda, Brazil, Italy and South Africa. Added trial physician for Brazil. Added Trial virologist for Uganda. Changed Principal Investigator for Mtubatuba. Added Kate Lee under trial management and co-ordination.

• **Section 2.1, 5.3.1, 9.1.1, 9.1.2.** Added participation of Italy, Brazil and Uganda throughout protocol.

• **Section 2.1, 4.3.1, 5.5, 9.3, 10.2.** Change primary outcome by adding 'or initiating late treatment (LTX)'.

• **Section 4.3.2.** Add secondary outcome 'Time from randomisation to reaching a CD4 of <350 cells/µl measured on 2 consecutive occasions not more than 4 weeks apart (not within first 3 months of trial entry)'.

• **Section 4.7.** New section added on mitochondrial toxicity sub-study.

• **Section 4.8.** Cap of one-third of patients randomised under PHI criterion 3 (detuned assay)

• **Section 5.1.** Add to end of first sentence 'with the exception of the genotypic resistance test which may not be available before randomisation'.

• **Section 5.4.** 'Results of the clinical history…etc' add to end of sentence 'and hypertension'.

• **Section 5.4.** Add to list of procedures (h) Trial specific immunology and virology samples when indicated in schedule 5.6 and (i) Blood for plasma storage

• **Section 5.5.** Resistance mutations will be interpreted according to current Stanford database algorithms instead of IAS guidelines.

• **Section 5.5, 5.6, 6.1.1.** All sites will store plasma at every visit (either from a separate sample or from the viral load blood) not just those not using the Bayer viral load assay.

• **Section 5.6.** The adherence questionnaire only needs to be completed during PTX.

• **Section 5.6, 6.2.1.1, 6.2.1.2.** Change collection tubes for Immunology samples from Sodium heparin to ACD and state that plasma will also be stored.

• **Section 6.2.** Australia not participating in the Immunology research work, only the UK.

• **Section 6.2.1.2.** Rename section as 'sites outside UK'.

• **Section 9.4.** New section added on 'Hepatitis B co-infection and use of short courses of ART'.

• **Appendix 7.** Added list of trial co-investigators and coordinating centres.

3. Revised protocol (number 3.0 date 19/02/07) with amendments to number 2.0 date 31/03/06

- **Renaming of "Section 4.6 Blood Pressure Subanalysis" to "4.6 Substudies" and "4.6.1 Blood Pressure Substudy"**
- **Renaming of "Section 4.7. Mitochondrial Toxicity Substudy" to "4.7 Subanalyses" and "4.7.1 Mitochondrial Toxicity Subanalysis"**
- **Section 4.7.2** New section on Effect of ART on Lipids sub-analysis
- **Section 4.7.3** New section on HIV Evolution sub-analysis

4. Revised protocol (number 4.0 date 19/06/07) with amendments to v3.0 date 19/02/07

- **Section 4.10** New section on the co-enrolment of Spartac patients on other studies.
- **Section 5.5 and 5.6** Added that Hepatitis B and C will be tested annually

- **Patient Information Sheet** Added that Hepatitis B and C tests will be done annually

5. Revised protocol (number 5.0 date 20/02/08) with amendments to v4.0 date 21/05/07

- **Page 3:** New Site and PI added
- **Page 4: Trial management and co-ordination** alteration of the detail of 2 members in this team
- **Page 11:** Spain added under list of countries trial is involved with
- **Section 4.10.4** new co-enrolment study added: First Consultation with a Health Professional During HIV Seroconversion
- **Section 4.10.5** new co-enrolment study added: The UK Register of HIV Seroconverters
- **Section 5.2** addition of the collection of baseline smoking status
- **Section 5.3.1** addition of Spain to the section
- **Section 5.4** addition of the collection of supplemental diagnoses during follow-up
- **Section 7.6** new section added: Supplemental Diagnoses
- **Section 9.1.1**: Spain added to section
- **Page 55:** Change in St Mary's, Durban and Italy Co-Investigators

6. Revised protocol (number 6.0 date **********) with amendments to v5.0 date 20/02/08**

- **Page 3:** Dr Paula Gama replaces Dr Monica Merçon as Trial Physician in Brazil. Update of contact details for trial physician and immunologist
- **Page 4:** Trial management and co-ordination alteration of the detail of 1 member in this team. Update of contact details for trial virologist
- ***Section 4: Sections 4.6 to 4.93*** the order of these paragraphs has been changed.
- **Section 4.2.3** The correction of a typographical error "all patients will be followed up for a minimum of **3.5 years**"
- **Page 56 & 57:** Updated contact details and name of trial investigators
- **Appendix 8:** Added patient information leaflet and consent form for the long-term storage of samples

2 SUMMARY

2.1 ABSTRACT

This protocol describes a randomised controlled trial comparing 3 different strategies of intervention in primary HIV infection (PHI). The primary outcome measure will be time to CD4 cell count <350 cells/µl (excluding counts in the first 3 months after diagnosis) on 2 consecutive occasions not more than 4 weeks apart or to initiating late treatment. Intervention at PHI is termed PTX (primary treatment) to distinguish it from late treatment (LTX), which may be administered according to local HIV treatment guidelines when indicated.

PHI is defined as documented HIV seroconversion through one or more of the following criteria:

- HIV positive antibody test within 6-months of an HIV negative antibody test (randomisation must take place within 6 months of previous negative test)
- HIV antibody negative with positive RT-PCR
- Test “incident” at low level (<0.6) using detuned assay (must be subtype B)
- Equivocal HIV antibody test supported by a repeat test within a 2-week period showing a rising optical density
- Clinical manifestations of symptomatic HIV seroconversion illness supported by antigen positivity and <4 bands positive on Western Blot

Eligible individuals must have reached the age of consent in their country for participating in a clinical study and satisfy one or more of the above criteria, and must be capable of giving written informed consent. Patients must fulfil these eligibility criteria before they can be screened. 360 individuals will be recruited over an 18-month period and randomised into one of three arms:

A. Long course combination antiretroviral therapy (LCART) for 48 weeks
B. Short course combination antiretroviral therapy (SCART) for 12 weeks
C. No antiretroviral therapy

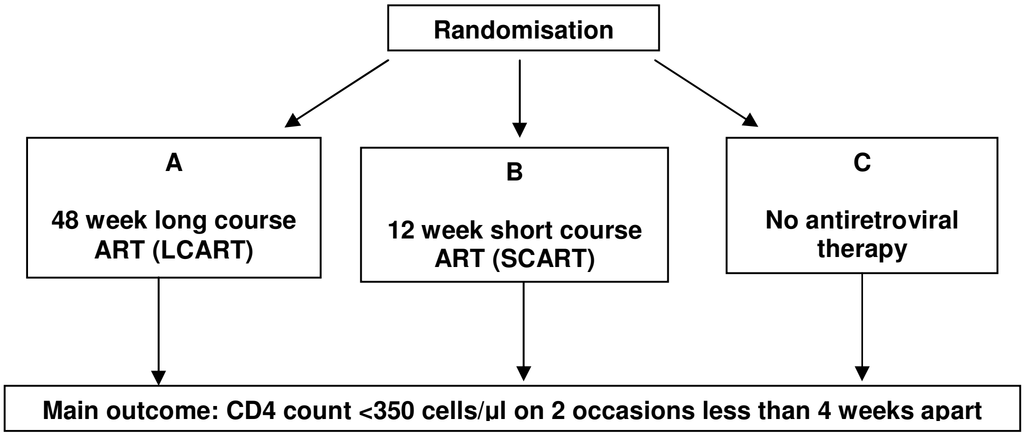

Following this intervention at PHI, all patients will cease PTX and will be followed according to the trial schedule (see 5.6). For Arm B, it is anticipated that, when ART is stopped at week 12, HIV RNA may still be detectable for a number of patients. ART should, however, be stopped at week 12 regardless of HIV RNA level. If disease

progression necessitates treatment, ART will be introduced according to the local standards of care. A decline in CD4 cell count to below 350 cells/µl during the first 3 months following randomisation will not be included in the endpoint analysis due to natural variability in CD4 counts close to HIV seroconversion.

For intolerance attributed to an individual drug, another licensed drug may be substituted where considered appropriate in accordance with local treatment guidelines.

The trial will be conducted in the following countries: South Africa, Australia, Italy, Uganda, Brazil, Spain, UK and Ireland. In Australia, Italy, Spain, the UK and Ireland all licensed drugs for the purposes of the treatment arms A and B of the study will be provided from within the local drug budgets, in accordance with national guidelines. In South Africa, Brazil and Uganda, drugs for patients at PHI will be provided through the study. Timing and provision of LTX will be determined according to local standards of care.

3 BACKGROUND

3.1 INTRODUCTION

The early events of HIV infection are likely to be important to the later course of the disease, and may be amenable to intervention. After mucosal exposure to HIV-1, virus is sequestered in regional lymph nodes until a threshold is reached within 2-6 weeks and viraemia occurs. This is termed primary HIV infection (PHI). Following PHI, virus is disseminated within days throughout the body[1,2,3] and seeds local, peripheral and distal reservoir sites. PHI is associated with a rise in plasma viral burden, which may be accompanied by either symptoms of a viral illness- 'seroconversion illness', immunosuppression[4,5,6] or, more often, is clinically asymptomatic. The initial viraemic peak falls to a “steady state” or set point within several months of infection, the level of which varies considerably between individuals and is predictive of prognosis[4,7]. At the same time, there is a vigorous HIV specific immune response involving cell mediated immunity, CD8+[8-17] and CD4+[18-20] specific responses, in addition to antibody production[21], all of which are believed to play an important role in controlling the initial plasma viraemia.

In order to generate effective CD8+ responses, it is believed that CD4+ mediated T-cell help is essential[12,22,23]. In particular, CD4+ mediated T-cell help is crucial for the effective priming and preservation of both HIV-specific antibody and CD8+ cytotoxicity responses[24,25]. It is clear that the principal immunological deficit in HIV-1 infection is the loss of HIV-specific CD4+ T-helper cell number and function[25,26]. Whilst HIV specific CD4+ T-cell helper responses are detectable in most individuals at seroconversion, the majority fail to mount robust HIV specific CD4+ T-cell responses after resolution of PHI[17,27]. Furthermore, despite broad immunological reconstitution following the introduction of antiretroviral therapy (ART) in chronic HIV infection, these HIV specific CD4+ T-cell responses are not usually recovered[27]. Indeed, HIV-specific CD4+ T-cell helper responses are not detectable in the majority of chronically infected subjects as assessed by HIV-specific lymphoproliferation[28].

However, using methods for the direct *ex vivo* quantification of antigen specific CD4+ T-cells, more recent reports have shown that HIV-specific CD4+ T-helper cells can be detected in most individuals with active infection[18,22]. These responses, however, decline with prolonged viral suppression. The strongest HIV- specific CD4+ T-cell responses using this technology are detected in patients with slow rates of clinical progression (long-term non-progressors) [29,30], and in patients treated with combination antiretroviral therapy (ART) at PHI[31-34.] If ART is commenced within weeks of acquisition of PHI, then these vigorous HIV specific CD4+ T-helper responses can be preserved[34,35]. These data are supported by animal studies that have shown that the earlier the therapeutic intervention the more effective the preservation of HIV-specific immunity associated with a consequent delay in disease progression[36,37]. However, in humans the time when treatment can be started is inevitably later than can be achieved during experimental infection. With the use of molecular diagnostic assays, early infection can now be more clearly defined and detected, prior to the development of an antibody response, so allowing earlier intervention.

Most studies have used at least one year of continuous therapy in PHI[38,39,40]. In the current climate of more conservative use of ART arising from the direct consequences of drug

toxicity, even in primary infection[41], adherence to complex regimens and the emergence of drug resistance, the possibility of a short course of therapy at PHI is an attractive option.

The aim of early intervention is to preserve HIV-specific CD4+ T-cell responses from HIV-induced lysis in order to confer enhanced control of viral replication when therapy is subsequently discontinued[42-44]. More recent reports presented at the 10th Conference on Retroviruses and Opportunistic Infections, Boston (2003) have not substantiated the early findings where HIV-specific CD4 T-helper responses remain detectable and at high level after three years or more from primary infection despite early ART intervention and initially good preservation[45]. Several other groups have also reported inconclusive findings on the long-term effects, and no study has directly correlated enhanced clinical outcome with early ART in PHI[46-48]. It is, therefore, crucial to definitively address this issue by a long-term randomised study, which is powered to answer this question.

3.2 JUSTIFICATION FOR THE TRIAL

There has been no randomised clinical trial of the treatment of PHI with combination ART, and so no direct evidence exists for decision-making. However, it is now clear that long-term ART is often accompanied by problems of adherence to therapy, primarily a consequence of intolerance of drugs and serious side effects. In addition, long-term use of drugs where adherence is poor is associated with the development of viral drug resistance. Hence life-long treatment from PHI may not be a feasible option.

Cessation of suppressive therapy will most likely be accompanied by a viral rebound[49,50]. However, a controlled level of detectable viraemia may be associated with perseverance of vigorous immune responses as seen in long-term non-progressor patients[40,42]. Furthermore, the psychological effects of receiving a new HIV diagnosis, the initiation of long-term drug therapy and the necessary life style changes that accompany this, may make a shorter course more acceptable to the patients than long-term therapy. Most HAART regimens have been demonstrated to achieve an undetectable plasma viral load in the majority of patients by 32 weeks with a median time of 10-12 weeks[51,52] to reach this goal. Early therapy in PHI has been shown to reduce the viral doubling time in those subjects receiving treatment compared with those untreated and even when therapy was stopped the viral doubling time was 5 times slower than during acute infection[53,54]. However, other factors may determine the duration of early therapy in addition to viral load.

In this study the short course therapy of 12 weeks (SCART) was chosen based on data from a pilot study undertaken using short course therapy during PHI[51]. By 12 weeks on HAART we anticipate >65% of adherent PHI patients will have plasma viral loads <50 copies/ml. For the long course ART arm (LCART) of 48 weeks on PTX we expect all patients to have experienced a period of undetectable plasma viraemia.

3.3 PILOT STUDY

A pilot study was conducted at St. Mary's hospital in 100 PHI patients of whom 84 initiated a short course of antiretroviral therapy of 12 weeks (initially Combivir/Nevirapine n=29, later Trizivir/Efavirenz n=35 or Combivir/Kaletra n=20). Patients not achieving a viral load below 50 copies/ml at week 12 continued to take therapy until a viral load below 50 copies/ml was achieved. The study demonstrated the successful preservation of HIV specific CD4+ T-cell responses to HIV in treated patients at 24 weeks of ART[51].

SCART was both acceptable and tolerable. Side effects were mild and infrequent; 3 patients developed rashes, all treated with anti-histamine drugs and none required cessation of therapy. One developed zidovudine induced anaemia and did stop treatment 2 weeks earlier than scheduled by the protocol, and one had a transient rise in ALT not necessitating interruption of therapy. 4/29 of those treated with Combivir/Nevirapine dropped one component due to side effects. 92/100 tested had wild type virus genotyped at baseline, whilst 2 had evidence of mutations associated with multi-drug resistant virus, 2 had mutations in Reverse Transcriptase gene and 1 had mutations in protease at baseline. No patient developed drug resistance (as defined genotypically) from short course treatment.

All individuals taking therapy achieved an undetectable plasma viral load <50 copies/ml irrespective of initial plasma viral load, with median time to achieving an undetectable plasma viral load being 10 weeks (range 4-32 weeks). Immunological studies from this pilot have identified preserved HIV specific CD4+ T-helper cellular immunity, in agreement with previously published studies and animal models.

However, 3/16 untreated patients in this study also showed preservation of HIV-specific CD4+ T-cells. It is clear that only a randomised study can reliably assess the efficacy of intervention at PHI.

3.4 CHOICE OF DRUG COMBINATION

The goal of therapeutic intervention at PHI is to provide rapid reduction of viral replication so as to protect HIV-specific CD4+ T-cells from destruction. The therapeutic regimen chosen must be as tolerable as possible in order to prevent stopping during a critical period of rapid viral replication. The choice of drug combination, therefore, should take into account short-term side effect profiles, pill burden, dietary restrictions, and tolerability. In the pilot study we compared the following combinations: Trizivir/Efavirenz, Combivir/Kaletra and Combivir/Nevirapine. There was no statistical significant difference between the groups in the time to <50 copies/ml. There were more side effects in the Trizivir/Efavirenz group, however, leading to 12% of patients having to stop or change treatment. Therefore, based on tolerability, we recommend Zidovudine/Lamivudine (as Combivir) plus Lopinavir/low dose Ritonavir (Kaletra) be used in the SPARTAC study.

4 GENERAL PLAN OF THE TRIAL

4.1 OBJECTIVES

4.1.1 Primary

To determine the effect of two anti-HIV treatment schedules of limited duration in PHI on the rate of CD4 cell decline and, consequently, on the time to initiating long-term anti-HIV therapy.

4.1.2 Secondary

To evaluate the effect of different durations of treatment during PHI on HIV-specific immune response and disease progression.

4.2 DESIGN

4.2.1 Randomisation and allocated treatment

Participants will be randomly allocated in a 1:1:1 ratio at trial entry to start one of the regimens of open treatment with:

- Arm A: Long course combination antiretroviral therapy (LCART) for 48 weeks
- Arm B: Short course combination antiretroviral therapy (SCART) for 12 weeks
- Arm C: No antiretroviral therapy

The regimen should be started, ideally, on the day of randomisation, or within 72 hours.

4.2.2 Choice of treatment

The PTX regimen to be used, should the patient be randomised to arms A or B, must be discussed with the patient beforehand and the chosen regimen must be recorded in the Trial Register before randomisation is allocated.

The recommended first line regimen for PTX regimen is:

- Zidovudine with Lamivudine, Lopinavir and low dose Ritonavir

If this combination of ART is considered unsuitable for reasons of intolerability, pill burden or possible adverse effects, the study physician can choose a different ART regime. The PTX regimen chosen should contain at least two nucleoside reverse transcriptase inhibitors with either a protease inhibitor (this is the recommended regimen) or a non-nucleoside reverse transcriptase inhibitor.

The combined regimen of Zidovudine, Lamivudine and Abacavir alone is not recommended

Factors, which may influence the choice of alternative PTX, include:

- History or evidence of HIV drug resistance from the potential source of HIV infection where available
- Previous history of other illnesses or infections such as chronic Hepatitis B/C
- Use of other prescribed medication(s) or recreational drugs
- ART regimen taken by partner/family
- Patient led preferences based on factual information and life style decisions
- Serum lipid profile

The exact drug combination chosen for each individual and any side effects or adverse events that may be associated with therapy must be clearly documented. PTX may also be modified due to adverse events, including substituting an individual drug with another not known to cause that side effect.

A genotypic resistance test will be requested on all patients at the screening visit to allow the results to be available to the clinician before randomisation so that PTX may be selected based on this information.

Upon stopping PTX for arms A and B, where an NNRTI has been used as part of the regimen, it is recommended that the NNRTI should be stopped at week 12 (arm A) or week 48 (arm B), but the other antiretrovirals need to be continued alone for 2 weeks. However, this time period should only be used for guidance, as the current lack of data does not allow for a definite optimum staggered stop for NNRTI used in HAART regimens. This staggered stopping of the NNRTI is based upon studies of pharmacodynamics of patients stopping the NNRTI with the aim of avoiding viral exposure to NNRTI monotherapy, and preventing the development of drug resistance[53]. For Arm B, it is anticipated that, when ART is stopped at week 12, HIV RNA may still be detectable for a number of patients. ART should, however, be stopped at week 12 regardless of HIV RNA level.

Individuals from all three arms will be followed up according to the schedule (see 5.6) with an additional initial safety visit for treated patients if required by local routine clinical practice. ART will be initiated according to local clinical practice guidelines, (e.g. when total CD4 count is between 200-350 cells/µl), or in the event of an opportunistic infection being diagnosed or symptomatic HIV disease.

4.2.3 Trial duration and population

It is planned to recruit 360 individuals internationally over a period of 18 months. The duration of intervention at PHI is a maximum of 48 weeks, and all patients will be followed up for a minimum of 3.5 years, making trial duration of 5 years.

4.3 EFFICACY MEASURES

4.3.1 Primary outcome

The primary efficacy endpoint will be the time from randomisation to reaching a CD4 of <350 cells/µl measured on two consecutive occasions not more than 4 weeks apart (not within first 3 months of trial entry), or to initiating late treatment (LTX).

4.3.2 Secondary outcomes

The secondary efficacy outcomes will include:

a. HIV-specific CD4+ and CD8+ T-cell responses at week 60;
b. Slope of CD4 decline;
c. Time from randomisation to virological failure of first regimen of late treatment (LTX) or death;
d. Development of drug resistance not present at baseline, before starting LTX or at week 120 whichever is earlier;
e. Development of an AIDS defining illness or death;

f. Time from randomisation to reaching a CD4 of <350 cells/µl measured on two consecutive occasions not more than 4 weeks apart (not within first 3 months of trial entry);
g. Time from randomisation to the initiation of late treatment (LTX);
h. Differences in blood pressure from randomisation at week 12 and week 48.

4.3.3 Criteria for AIDS
AIDS events are defined for the trial using criteria in Appendix 4

4.4 TOXICITY MEASURES

4.4.1 Primary
The primary toxicity endpoint is any grade 3 or 4 (clinical or laboratory) adverse events (whether expected or unexpected).

4.4.2 Secondary
The secondary toxicity endpoint is any serious adverse event (whether expected or unexpected)

4.5 ADHERENCE MEASURES

All participants taking therapy will fill out a self-reported questionnaire about missed pills including mistakes in their medication schedule (Appendix 5). Adherence measures will include the proportion of missed pills since last visit.

4.6 ELIGIBILITY AND EXCLUSIONS

4.6.1 Inclusion criteria

Patients will be eligible for screening if they:
- Have reached the age of consent in their country for participating in a clinical study
- Are confirmed PHI by at least one of following criteria:

1. HIV positive antibody test within 6-months of an HIV negative antibody test (randomisation must take place within 6 months of previous negative test)
2. HIV antibody negative with positive RT-PCR
3. Test "incident" at low level (<0.6) using detuned assay (must be subtype B) (there is a cap of one-third of randomised patients under this criterion)
4. Equivocal HIV antibody test supported by a repeat test within a 2-week period showing a rising optical density
5. Have clinical manifestations of symptomatic HIV seroconversion illness supported by antigen positivity and <4 bands positive on Western Blot

- Able and willing to give written informed consent

The generic Patient Information Sheet and Consent Form (Appendix 2) will be adapted to local needs and/or translated as required.

4.6.2 Exclusion criteria

Patients will not be eligible for screening if:
- Pregnant
- Unlikely to comply with protocol, and in particular adhere to therapeutic regimen

- Likely to use narcotics during the study period
- Antiretroviral therapy is indicated
- Antiretroviral therapy is contraindicated.

4.7 CONCOMITANT MEDICATION

If treatment is required for any other infections (such as tuberculosis) or other medical conditions, then local hospital policies, national HIV guidelines and drug information in the packaging information should be followed.

4.8 SUBSTUDIES

4.8.1 Blood pressure sub-study

A sub-analysis will be undertaken to explore the association between blood pressure and HIV and/or anti-retroviral therapy. The aim is to determine whether anti-retroviral therapy is associated with raised blood pressure and whether any such rise may be temporary and normalises once therapy is stopped. To standardise the blood pressure measurements a British Hypertension Society approved, semi-automatic device, the OMRON 705-CP will be used to measure the patients' blood pressure. At each scheduled visit 3 seated measurements are made and recorded on the CRF. The study has 80% power at the 5% level to detect a 5mmHg increase in systolic blood pressure in the treated groups. All patients randomised into the trial will be included in this sub-analysis. Information on medication for anti-hypertension and alcohol consumption will be collected on the CRF as these are known to affect blood pressure.

4.9 SUBANALYSES

4.9.1 Mitochondrial toxicity sub-analysis

Mitochondrial toxicity in patients with HIV infection has been associated with the use of nucleoside analogues. This sub-study will be undertaken to quantify mitochondrial DNA copy number in patients with PHI, following short course HAART during PHI and 3 months after completion of this intervention. The aim is to determine whether mitochondrial depletion is present in PHI or during short course HAART at PHI, and if so the severity of this depletion and whether it recovers after therapy. DNA will be extracted from PBMCs at 3 time points: screening, cessation of HAART (week 12 for SCART/week 48 for LCART), 12 weeks after stopping HAART (week 24 for SCART/week 60 for LCART). DNA copy number will be determined by 'real-time' PCR. This sub-study will be undertaken on a sample of 20 UK patients only.

4.9.2 Effect of ART on lipids sub-analysis

Protease inhibitors (PIs) have long been associated with elevating plasma lipids (in particular, Kaletra). SPARTAC provides a unique opportunity to assess the effect of PIs on ART naïve individuals and the time frame for lipid changes to resolve upon ART cessation. Whether the effect of ART initiated at PTX causes similar elevations in lipids to those initiating ART at LTX will also be studied. To investigate these issues, the differences in median lipid values between the three arms at week 12, 48 and annually will be examined, along with comparing differences in the rate of change over time, adjusting for baseline lipid measurements (at week 0) and potential confounders (e.g. sex, age, risk group). In addition there will be comparisons of lipids after cessation of PTX with values while on PTX and of differences in median lipid values between the 3 arms for those initiating LTX at fixed time points with rates of change adjusting for CD4 at LTX initiation and potential

confounders.

4.9.3 HIV evolution sub-analysis

Mutations in HIV-1 that facilitate escape from the HLA Class I mediated immune response are well documented in individual patients. This analysis aims to characterise the development of these polymorphisms at a 'population' level, exploring their impact on viral evolution and the host immune response. The aim is to define the evolution of HLA-associated genetic diversity from the time of infection until established chronic infection and to interpret characteristic 'signature' patterns of HIV-1 sequence variation which appear to be determined by population HLA frequencies. No extra samples are required and extra data will be obtained from viral sequences at 6-monthly to 1 year intervals post-seroconversion. Plasma for this is available from the supernatant from the collection of CD4 T cells at the Peter Medawar Building, Oxford. HLA Class I typing data will be required from pre-existing databases. Sequence data from the *Pol, Gag* and *Nef* genes of HIV-1+ve patients in SPARTAC will be analysed for polymorphisms and analysis will be done to identify associations between each HLA Class I molecule and polymorphisms at every codon for each longitudinal time-point (seroconversion, year 1, year 2, etc). The analysis will reveal sites within the genome that are under the strongest selection pressure shortly after infection, those HLA Class I molecules that are able to contain viral infection and those which are most susceptible to immune escape.

4.10 CO-ENROLMENT OF SPARTAC PATIENTS ON OTHER STUDIES

4.10.1 Highly exposed persistently seronegative uninfected individuals (HEPS) study

This is a study separate from Spartac that has been given its own ethics approval (approved by the St Mary's Research Ethics Committee on 16th August 2006, REC reference number 04/Q0403/15) and will approach patients (at St Mary's Hospital only) enrolled in Spartac to participate in this study.

Study background: Despite repeated exposure certain individuals appear to be protected from established infection with HIV-1. These individuals have been defined as exposed uninfected (EU). The aim of the study is to investigate whether these EUs (or HEPS) have low levels of HIV-1 present in either plasma, PBMCs, rectal tissue and whether there significant differences between the phenotype and/or function of the lymphocytes infiltrating the rectal mucosa of HEPS compared to their HIV+ve partners. A small number of individuals currently participating in the SPARTAC study have HIV negative partners who have been exposed to their partner's HIV but not become infected. These couples will be enrolled in an on-going HIV serodiscordant couples study whereby they will attend clinic once at a separate time to their SPARTAC visit. During this visit participants will complete a sexual behaviour questionnaire, have 20mls of blood taken and a rigid sigmoidoscopy with rectal biopsy carried out. Virus from both blood and rectal tissue will be extracted and co-receptor usage determined. In addition an ultra-sensitive HIV DNA PCR and HIV specific immune tests will be carried out and results compared to their HIV negative partner.

4.10.2 HIV/ hepatitis C (HCV) co-infection study

This is a study separate from Spartac that has been given its own ethics approval (by the Riverside REC on 14th March 2005, REC reference number 05/Q0401/17) and will approach patients (at St Mary's Hospital only) enrolled in Spartac to participate in this study.

Study background: Participants (at St Mary's Hospital) with HIV and HCV co-infections are

eligible for the St Mary's co-infection cohort, the aim of which is to determine the diversity of HCV in HIV co-infected patients over time. Specifically, the rate of HCV evolution by sequence and phylogenetic analysis of the viral envelope hypervariable region, the role of viral escape and whether it's driven by T cell responses, and the specificity of T cell responses in chronically infected individuals compared to those who have cleared the infection will be studied. HLA typing will be known by standard PCR SSCP methodology for all patients, with immunological assays carried out at 2-4 time points following HLA typing: before infection with HCV, at the time of diagnosis of acute HCV, and 1-2 annual time points thereafter. 1 vial of cells (2 millions PBMCs) will be used from each time point for these assays. ELISpot assays to identify interferon-gamma secreting T cells reacting against a range of HCV-derived antigens will map responses to single peptides and measure cytotoxic T cell responsiveness to variant peptides, while MHC class I peptide "tetramers" will assess the frequency and phenotype of such responses. The date of HCV infection is established by nested RT-PCR amplification of a conserved region of the HCV genome, and the evolution of diversity will be addressed by nested PCR amplification using specific primers of the HCV E2 Hypervariable region, along with other parts of the HCV genome.

4.10.3 ALTAIR study

This is a study separate from Spartac that has been given its own ethics approval (by the Oxfordshire REB B on 1st February 2007, REC reference number 07/Q1605/6, EudraCT number 2006-004476-12) and will approach patients (from Altair /Spartac sites only) enrolled in Spartac to participate in this study.

Study background: Altair is a randomised, open-label, 96-week study comparing the safety and efficacy of three different combination antiretroviral regimens as initial therapy for HIV infection. The primary objective of this study is to compare the virological efficacy, as measured by the timeweighted mean change from baseline plasma HIV-RNA, and safety, of three strategic regimens of initial antiretroviral therapy (ART) containing a fixed dose formulation of tenofovir and emtricitabine, with either efavirenz or ritonavir boosted atazanavir or zidovudine plus abacavir. (Primary comparisons are regimen I versus II and I versus III as described below).

I. tenofovir (TDF) + emtricitabine (FTC) + efavirenz (EFV)

II. tenofovir (TDF) + emtricitabine (FTC) + ritonavir/atazanavir (r/ATV)

III. tenofovir (TDF) + emtricitabine (FTC) + zidovudine (ZDV) + abacavir (ABC)

This is in order to assess whether the research hypothesis - in treatment-naïve HIV-infected subjects, combination antiretroviral therapy including efavirenz combined with tenofovir and emtricitabine will offer non-inferior antiretroviral efficacy over 48 weeks, compared to either atazanavir boosted with ritonavir combined with tenofovir and emtricitabine or tenofovir and emtricitabine combined with zidovudine and abacavir, as assessed by change from baseline plasma HIV-1 RNA viral load – is accurate.

4.10.4 First consultation with a health professional during HIV seroconversion

This is a study separate from Spartac that has been given its own ethics approval (by the St Mary's REC on 1st June 2007, REC reference number 07/Q0403/61) and will approach patients enrolled in Spartac to participate in this study.

Study background: The aim of this study is to determine if health practitioners in the community may be missing out on the possibility for early medical interventions when not including the potential for acute HIV infection in their differential diagnosis of patients who are at risk. This will be examined via a retrospective, observational comparison of those patients, all of whom have recently gone through HIV seroconversion, who had or had not

disclosed their sexual orientation at the first consultation with a health professional during what they now know to be their acute HIV seroconversion.

The participants of this study will be approached and consented to a one-time, 20-item questionnaire to complete, which details the chronology of this first consultation with a health practitioner, including the time from the onset of symptoms to their presentation to the health practitioner, consultation process and outcome, follow-up care or further presentations to a health practitioner, any referrals, decision to test for HIV as well as location of clinic when testing for HIV and disclosure of HIV status to GP since diagnosis. Specific blood results, from the patient's first visit, including Viral Load, CD4, Hepatitis A, B and C serology as well as any concurrent sexually transmitted infections at presentation (i.e., Gonorrhoea, Chlamydia, Syphilis, LGV, Non-specific Urethritis, etc) will also be gathered. No new clinical investigations will be obtained from the participant, only the participant's previous laboratory results from their first visit This information will be compiled and quantitatively analysed to construe and compare the percentages between two groups of individuals; those who had or had not disclosed their sexual orientation with a health professional during what they now know to be their acute HIV seroconversion. The gathering of the data of any concurrent sexually-transmitted infections in these participants will be used to highlight the potentially enhanced transmissibility of HIV during acute HIV infection.

4.10.5 The UK register of HIV seroconverters

This is an observational study separate from Spartac that has been given its own ethics approval (by the South Birmingham REC on 12th November 2004, REC reference number 04/Q2707/155) and will approach patients enrolled in Spartac to participate in this study once they are no longer attending trial visits to allow data from their routine follow up to be collected.

Study background: The UK Register is a national cohort of individuals whose time of HIV seroconversion can be reliably estimated. The study aims to assess temporal changes in the time from HIV seroconversion to AIDS and death (i.e. effectiveness of therapy at the population level), to examine the effect of time since infection on virological, immunological and clinical response to therapy, and to assess the prevalence of transmitted drug resistant HIV and its long-term consequences. As well as becoming a valuable tool in the clinical management and counselling of infected individuals, the results of such investigations have been crucial in estimating the future burden of HIV disease on public health.

Spartac participants in the UK only will be given the patient information sheet by the clinic nurse and asked to enrol in the study after giving informed signed consent. As an observational study there are no study visits to attend. Clinical staff caring for consented patients are asked to register them by completing the study initial registration form. Clinic staff are also requested to complete an annual follow-up form for registered patients. At the initial registration and once a year the patient is asked for a 10ml blood sample for central storage.

5 PROCEDURES

5.1 SCREENING

Potentially eligible individuals can be screened within a 1-4 week period before trial entry but results from screening must be available for randomisation with the exception of the genotypic resistance test which may not be available before randomisation. All patients must be given adequate information about the trial together with a **Patient Information Sheet** (Appendix 2) and be given an opportunity to ask questions about the trial. **The patient and study doctor must both sign 2 copies of the main study consent form (1 copy to be given to the patient, 1 copy kept in patient's clinic notes) before any study procedures are carried out. Additional specific consent is requested for the genetic studies on a separate consent form which the patient and doctor need also to sign 2 copies of as with the main study consent form.**

All individuals screened must have their name, date of birth and clinic number recorded in the Trial Register. The register must be stored by the investigator in a secure place only accessible to appropriate clinical staff. If an individual is not subsequently randomised the reason should be recorded in the register.

Screening will include:

a) Recording the clinical history, including HIV test results
b) Physical examination
c) Pregnancy test in women of childbearing potential
d) Full blood count (haemoglobin, white cells, neutrophils, lymphocytes and platelets)
e) Biochemistry (potassium, creatinine, bilirubin, AST or ALT, alkaline phosphatase, amylase or lipase, and glucose [record as fasting or non-fasting])
f) T lymphocyte subsets (total lymphocytes, percent and/or absolute CD3/CD4/CD8)
g) Viral load measurements
h) Hepatitis B and C status
i) Syphilis
j) TB screen if indicated
k) Trial specific virology samples to be collected
l) Genotypic resistance test

Results of the clinical history and evaluations are to be entered on a case record form.

5.2 TRIAL ENTRY (WEEK 0)

To proceed, all individuals must have fulfilled the eligibility criteria by the results of evaluation at screening.

Confirm that the individual continues to consent to enter the trial. If the participant has agreed to their GP being informed about participation, a letter should be sent at this point (sample letter Appendix 3).

Entry procedures will include:

a) Recording the clinical history since screening, including AIDS events, presence of non-HIV related adverse events, and current use of concomitant medication for opportunistic infections and anti-hypertension.
b) Physical examination, including weight, height and blood pressure.
c) Full blood count (haemoglobin, white cells, neutrophils, lymphocytes & platelets),

d) Biochemistry (potassium, creatinine, bilirubin, AST or ALT, alkaline phosphatase, amylase or lipase, cholesterol and triglycerides and glucose [fasted]).
e) T lymphocyte subset (total lymphocytes, percent and/or absolute CD3/CD4/CD8)
f) Plasma HIV RNA by the most sensitive assay available at the local laboratory
g) Trial specific Immunology samples to be collected
h) Blood for plasma storage
i) Smoking status at baseline (this will be collected retrospectively)

5.3 RANDOMISATION

5.3.1 Sites in UK, Ireland, South Africa, Italy, Brazil, Uganda and Spain

To randomise a patient contact the **MRC Clinical Trials Unit (tel. 44 20 7670 4766/ fax 44 20 7670 4814).** Eligibility will be checked by the CTU. Essential information required will be the trial number, clinic number, date of birth, whether they fulfilled eligibility criteria for PHI through having a negative HIV antibody test (within last 6 months or currently with PCR positivity) or through another criterion. Also required is the PTX regimen that will be used if they are randomised to arm A or B. The randomised intervention will be assigned to the trial number which should be used on all subsequent case record forms and must accompany all specimens. If the participant withdraws from the trial this number must never be re-used for another individual. Enter the trial number, date of randomisation, the allocated intervention and the date of first prescription of PTX if allocated to arms A or B in the Trial Register.

5.3.2 Sites in Australia

Randomisation will be carried out by the National Centre in HIV Epidemiology and Clinical Research (NCHECR). To randomise a patient contact the **NCHECR (tel: 61-2-9385 0900/ fax 61-2-9385 0920 for the attention of Pat Grey)**. Eligibility will be checked by NCHECR. Essential information required will be the clinic number, date of birth, whether they fulfilled eligibility criteria for PHI through having a negative HIV antibody test (within last 6 months or currently with PCR positivity) or through another criterion. Also required is the PTX regime that will be used if they are randomised to arm A or B. The randomised intervention will be assigned to the trial number which should be used on all subsequent case record forms and must accompany all specimens. If the participant withdraws from the trial this number must never be re-used for another individual. Enter the trial number, date of randomisation, the allocated intervention and the date of first prescription of PTX if allocated to arms A or B in the Trial Register.

5.4 ROUTINE FOLLOW-UP

Follow-up will be at weeks 4, 12, 16, 24, 36, 48, 52, 60 then every 12 weeks until the trial closes and will include **(see visit schedule, section 5.6)**:

a) Clinical history since last assessment, including the time of the last doses of trial drugs taken, AIDS events, presence of non-HIV related adverse events, use of concomitant medication for opportunistic infections and anti-hypertension, and occurrence of pregnancy.
b) Physical examination including weight and blood pressure if indicated by medical history.
c) Patient adherence questionnaire.
d) Full blood count (haemoglobin, white cells, neutrophils, lymphocytes and platelets)

e) Biochemistry (potassium, creatinine, bilirubin, AST or ALT, alkaline phosphatase, amylase or lipase, cholesterol and triglycerides and glucose - this should be a FASTING specimen, wherever possible).
f) T lymphocyte subsets (total lymphocyte, CD3, CD4 and CD8)
g) Local plasma HIV RNA by the most sensitive assay available at the local laboratory.
h) Trial specific immunology and virology samples when indicated in schedule 5.6.
i) Blood for plasma storage
j) Hepatitis B and C status when indicated in schedule 5.6
k) Collection of supplemental diagnoses (see section 7.6)

Patients randomised to Arms A and B will additionally attend for a safety visit at week 2 or according to normal routine care for patients on HAART.

Results of the clinical history, trial treatment and other anti-retroviral drugs, concomitant medication for opportunistic infections and hypertension, non-HIV related adverse events and laboratory results should be entered on a separate case record form. AIDS events or death should also be recorded on case record forms as should serious adverse events.

There is no restriction on the investigator including extra assessments, including blood tests for monitoring, at other times. Any additional results for haematology, biochemistry (including abnormal results for non-protocol tests), T lymphocyte subsets or local plasma HIV RNA should be recorded on case record forms.

5.5 PROCEDURES FOR ASSESSING EFFICACY

The primary end point of the trial will be met when the patient's absolute CD4 cell count falls below 350 cells/µl on two consecutive occasions not more than 4 weeks apart or when the patient initiates late treatment. Any such CD4 values measured within the first 3 months of randomisation, however, will be ignored.

HIV-specific CD4 T-cell frequencies will be determined by interferon-γ ELISPOT analysis. Results are expressed as specific spot forming cells (SFC) per 10^6 CD8-depleted Peripheral blood leukocytes (PBL).

Virological failure is defined as failure to suppress HIV RNA to below 50 copies/ml within 6 months of initiating PTX or, having suppressed within the first 6 months, experiencing a rebound in HIV RNA to >400 copies/ ml

Resistance mutations will be interpreted according to the current Stanford database algorithm on key mutations associated with reduced susceptibility to antiretroviral drugs.

AIDS will be defined according to the criteria in Appendix 4.

Although most sites will use the Bayer (b-DNA) assay for quantifying HIV RNA, some sites will be using other assays. In order to have comparable HIV RNA measurements at the same time points, for those sites not using the Bayer assay, one aliquot of the EDTA plasma stored at each visit be tested centrally at a later time point.

5.6 SCHEDULE OF VISITS AND INVESTIGATIONS

**Weeks**	**Visit 1 Screening**	**Visit 2 Randomi-sation**	**Visit 3 Week 4**	**Visit 4 Wk 12**	**Visit 5 Wk 16**	**Visit 6 Wk 24**	**Visit 7 Wk 36**	**Visit 8 Wk 48**	**Visit 9 Wk 52**	**Visit 10 Wk 60**	**Twelve weekly**
Completion of case record form	X	X	X	X	X	X	X	X	X	X	X
Adherence questionnaire*			X	X	X	X	X	X			
Physical examination	X**	X	X**	X**	X**	X**	X**	X**	X**	X**	X**
Urine pregnancy test	X										
Full blood count	X	X	X	X	X	X	X	X	X	X	X
Biochemistry	X	X	X	X	X	X	X	X	X	X	X
T-cell counts & viral loads	X	X	X	X	X	X	X	X	X	X	X
Hepatitis B & C status	X										X (Annually)
Syphilis serology	X										
TB screen	X**										
Plasma store (4ml EDTA or from viral load blood)		X	X	X	X	X	X	X	X	X	X
Virology bloods (8ml clotted)	X			X	X	X			X	X	X (Annually)
Virology bloods (20ml sodium heparin preservative-free)	X								X		X (Annually)
Immunology bloods (40ml ACD)		X		X	X	X		X	X	X	X (Annually)
Genotypic resistance test (6ml EDTA)	X				X (arm B)	X ***			X (arm A)	X	X (Annually)

* for patients on PTX ** If indicated on history *** arm A if HIV RNA is detectable

6 VIROLOGICAL AND IMMUNOLOGICAL SPECIMENS AND STUDIES

6.1 VIROLOGY

Blood samples will be taken from all subjects at screening, and stored at -80ºC or lower. Viral isolations will be attempted from all subjects on day of screening, by means of the published WHO protocol for primary HIV isolation. All successful viral isolates, as defined by detectable p24Ag by day 28 of culture, will be aliquoted and stored in liquid nitrogen. All isolates will be studied for:

- viral subtype in *pol*, as determined by population sequencing of RT and Pro;
- phenotype, as defined by the induction of syncytia in the MT2 cell line, and isolates will be classed as syncytium inducing (SI) or non-syncytial inducing (NSI);
- co-receptor usage, as defined by the ability of the isolates to grow in U-87 cells expressing CCR5 or CXCR4, or both;
- neutralisation susceptibility, as defined by the ability of the viral isolate to be neutralised in a primary PBMC assay by a panel of standard antibodies including B12, PO52, and SB62.

A sub-set of virus isolates will be studied longitudinally in order to ascertain the impact of ART on the emergence of viral neutralisation over time.

Plasma PBMCs will be stored from visit 1 of the protocol. For subjects who have been specifically consented to genetic studies on stored material, DNA will be extracted by standard procedures, in order to investigate host genetic polymorphisms. The polymorphisms which will be sought include: known CCR5, SDF-1 promotor and other chemokine receptor polymorphisms; drug efflux enzymes; HLA haplotype; other polymorphisms that may affect the course of HIV infection.

6.1.1 Local Laboratories

Local plasma HIV RNA assays

All plasma HIV RNA quantification will be undertaken in the local laboratory using the most sensitive routinely available assay at the time. As most clinical sites enrolling patients use the Bayer (Chiron bDNA) assay for viral load determination, this will be the standard assay for the trial. At sites where this is not feasible, an aliquot of the plasma stored at -80ºC or below at each visit as laid out in Schedule 5.6 will be re-tested at the Spartac central virology laboratory for viral load quantification at a later date. This is required in order to establish standardised viral load measurements for all trial patients.

Additionally, at least one aliquot of EDTA plasma taken separately or for HIV RNA quantification will be stored at each time point regardless of which viral load assay is used.

The Virology Group will advise on the need for quality assurance (QA) depending on the procedures already set up within each country.

6.1.2 Central Laboratories

<u>Research Samples</u>

A total of 34ml of bloods will be collected as follows: 8ml clotted blood for serum and 20ml of sodium heparin blood, which will be separated into plasma for storage and PBMCs. Virus isolation will be undertaken on the PBMCs and the remaining (about 10-15 million cells) will be stored in vapour-phase liquid nitrogen. An aliquot of serum from bloods taken on the day of screening will be stored.

Additionally, 6ml of EDTA blood is required for genotypic resistance testing. The 6ml EDTA bloods for weeks 16, 52, 60 and then annually, will be frozen at the Central Virology laboratories and a genotypic resistance test performed once the viral load measurement performed by the local laboratory on samples taken at the same time are known to be >50 copies/ml.

All bloods which are not being stored need to be sent to the relevant virology laboratory and are to arrive there by 12 mid-day, or as agreed with the laboratory, in order to ensure that the research work to be carried out on fresh samples is undertaken on the same day. These samples can be transported at room temperature.

6.2 IMMUNOLOGY

Five million cells will be set aside for an HIV specific CD4 ELISPOT assay as outlined in Schedule 5.6. We intend to perform a robust, reproducible assay of the HIV specific CD4 gag helper response in patients recruited at clinical centres where this assay can be applied. Because the assay is only reliable on fresh blood samples, only those patients recruited in the United Kingdom, will be suitable for this evaluation. The purpose of this assay is to provide an indication of the frequency of these antigen specific cells in patients before, during and after the intervention. These assays will test the hypothesis that combination antiretroviral therapy preserves this population of cells as compared to the attrition measured and recorded in those who receive no treatment at all.

6.2.1 Central Laboratories

6.2.1.1 UK sites

40ml of ACD blood will be collected at baseline and at all time points indicated in schedule 5.6. Fresh blood will be dispatched immediately at room temperature to the relevant laboratory, for receipt no more than 6 hours after drawing. From the baseline sample, DNA will be isolated and analysed for HLA class I and class II expression. Lymphocyte fractions will be isolated at all time points using Hypaque gradients. The freshly isolated lymphocyte samples will be assayed immediately for HIV-specific CD4 T-helper cell responses using interferon gamma secreting ELISPOT technology. Peptides will be derived from known immunodominant regions of HIV-1 epitopes for all clades of HIV-1 prevalent within the populations tested and adjusted for each patient depending on their HLA genotype.

Lymphocytes will also be stored at a temperature between -80ºC and -150ºC for later analysis of HIV-specific CD8 T-cell responses, using optimised peptide based interferon gamma secreting ELISPOT technology. It is anticipated that around 5-7 million cells per

tube (1-8 tubes) will be stored at each time point. Plasma will also be stored from this sample.

6.2.1.2 Sites outside UK

40mls of ACD blood be collected at baseline and at all time points indicated in schedule 5.6. Lymphocytes will be stored at a temperature between -80ºC and -150ºC for later analysis of HIV-specific CD8 T-cell responses, using optimised peptide based interferon gamma secreting ELISPOT technology. It is anticipated that around 5-7 million cells per tube (1-8 tubes) will be stored at each time point. Plasma will also be stored from this sample.

6.2.2 Local Laboratories

All CD3, CD4 and CD8 cell counts will be undertaken in the local laboratory using routine assays and procedures.

A quality assurance (QA) exercise will be undertaken on those laboratories which are not accredited under a recognised scheme. Trial immunologists will advise on the timing and detail of such an exercise.

7 ADVERSE EVENTS

7.1 DEFINITION

Adverse events are any adverse experiences whether or not considered to be drug-related. Adverse events may be expected or unexpected. These may include side-effects, injury, toxicity or sensitivity reactions, abnormal laboratory results and concurrent illnesses **but for this trial exclude those events known to be HIV-related.**

An expected, drug-related adverse event is one previously reported in the current summary of product characteristics for the drug.

Mild adverse events should be managed symptomatically and therapy should be continued at full dose:

Nausea	anti-emetics
Headache	simple analgesia
Mild rash	topical emollients, topical steroids (hydrocortisone 1%), anti-histamines
Diarrhoea	anti-motility drugs

Unexpected fatal or life-threatening events must be reported immediately to the regulatory authorities by the investigator

7.2 GRADING OF ADVERSE EVENTS

Criteria for grading laboratory and clinical events are listed in appendix 6.

7.3 SEVERE GRADE ADVERSE EVENTS

Severe adverse events are those which are **graded 3 or 4** by standard toxicity criteria in appendix 6. Some, but not all, grade 3 and 4 adverse events will also be "serious" by ICH GCP criteria below.

7.4 SERIOUS ADVERSE EVENTS (SAE) DEFINITION

An adverse event is considered to be reportable as a "serious" adverse event (SAE) by ICH GCP criteria if it:

- is fatal
- is life-threatening (**with an immediate, not hypothetical, risk of death at the time of the event**)
- requires in-patient hospitalisation or prolongs existing hospitalisation (**hospitalisation for elective treatment of a pre-existing condition is not included**)
- results in persistent or significant disability or incapacity
- is a congenital anomaly (i.e. the outcome of pregnancy involving a participant)
- is any other important medical condition*.

*Examples of conditions regarded as "any other important medical condition" include allergic bronchospasm requiring intensive emergency treatment, seizures or blood dyscrasias, which did not result in hospitalisation or development of drug dependency.

SAEs which begin before randomisation are not reportable. Any non-AIDS defining cancer should be reported as an SAE.

Note that some relatively low-grade events may fulfil the criteria.

7.5 REPORTING OF GRADE 3 & 4 AND SERIOUS ADVERSE EVENTS

All grade 3, 4 and serious adverse events should be recorded on case record forms regardless of the relationship to therapy. There should not be an unreasonable delay in reporting these events.

7.6 SUPPLEMENTAL DIAGNOSES

In addition to the collection of adverse events, the occurrence of supplemental diagnoses will be collected. This is a fixed list of events inclusive of:

1. Alcoholism/alcohol abuse
2. Avascular necrosis (AVN) of the bone
3. "Buffalo hump" (enlargement of dorsocervical fat pad)
4. Cancer (excluding lymphoma, Kaposi's sarcoma and invasive cervical cancer)
5. Cirrhosis
6. Congestive heart failure
7. Coronary artery disease (i.e., angina) requiring drug treatment
8. Coronary artery disease requiring an invasive or surgical procedure
9. Diabetes mellitus requiring dietary modification only
10. Diabetes mellitus requiring drug treatment
11. End-stage renal disease
12. Hepatic steatosis as evidenced by liver biopsy
13. Lactic acidosis
14. Myocardial infarction
15. Myocarditis
16. Pancreatitis
17. Pericarditis
18. Peripheral lipodystrophy (thinning of face, limbs, or upper trunk)
19. Peripheral vascular disease, including invasive or surgical procedures for vascular disease (excluding angina, arrhythmia, congestive heart failure, coronary artery disease, hypertension, and valvular disease)
20. Renal insufficiency
21. Significant increase in abdominal girth as subjectively reported by patient or as measured by clinician
22. Stroke
23. Unexplained breast enlargement as subjectively reported by patient or as measured by clinician

The presence of any of these supplemental diagnoses at screening and to date will be collected retrospectively on a case record form and any subsequent diagnoses should be reported to MRC CTU without an unreasonable delay in reporting.

8 WITHDRAWAL BY SUBJECTS

Participants may discontinue taking part in the trial at any time and for any reason, but

they should be asked, if they are willing to give a reason, and this reason recorded. All participants must be asked whether they agree that data from their routine care can subsequently be used to complete follow-up case record forms. If so the frequency of assessment should be 12 weekly where possible.

9 ANTIRETROVIRAL MEDICATION

9.1 DISPENSING

9.1.1 Australia, Italy, Spain, UK and Ireland

Drugs will be dispensed from hospital stock at all sites in accordance with local and national guidelines. No additional records will be required beyond normal practice.

9.1.2 South Africa, Brazil and Uganda

Drugs will be dispensed from study specific supplies. Each drug dispensed must be recorded on the study dispensing log.

9.2 MODIFICATION OF TRIAL TREATMENT

For both treatment arms A and B toxicity must be managed according to local recommendations and alternative antiretroviral drugs will be prescribed when indicated. Toxicities leading to drug interruption, discontinuation or dose reduction should be recorded on case record forms.

LCART arm at visit 6 (week 24) if plasma viral load is detectable a genotypic resistance test should be attempted on an additional sample taken at that visit. If evidence of drug resistance has developed since screening the PTX regime must be changed within 4 weeks of result and patient will be asked to attend for an additional visit. Adherence must be addressed at this point.

9.3 INITIATION OF LATE TREATMENT

If, after the first 3 months of study participation, a patient's CD4 cell count is below 350 cells/µl a second CD4 count must be performed within 4 weeks of that test and if the result of the second test is also below 350 cells/µl the study endpoint has been met.

Late treatment (LTX) can be commenced at any time after this point governed by local practice, the choice of drugs must be based on the available drug resistance results, the patient's and their doctor's preference and according to local standards of care. If a patient initiates late treatment before reaching CD4 below 350 cells/µl, the study endpoint is met when late treatment is initiated.

9.4 HEPATITIS CO-INFECTION AND USE OF SHORT COURSES OF ART

There have been reported cases of potentially dangerous exacerbations of chronic Hepatitis B after the withdrawal of Lamivudine used to treat Hepatitis B or as part of an ART regimen used to treat HIV in co-infected subjects. This has been reported to occur in approximately 16% of chronically co-infected HIV and Hepatitis B patients. It is usually of limited clinical significance but can rarely (5%) decompensate poor liver function and lead to incipient liver failure[56].

In order to avoid any potential problems with patients enrolled in Spartac we recommend that all individuals testing Hepatitis B surface antigen positive who are eligible for Spartac are allocated non- Hep B active ART. It is recommended that pre-randomisation the results of the Hepatitis B test is available to the clinician and ART MUST NOT include lamivudine FTC or Tenofovir. Zidovudine, Didanosine and Kaletra is recommended as an acceptable and safe regimen to employ for any eligible individuals with evidence of chronic Hepatitis B infection.

10 STATISTICS

10.1 METHOD OF RANDOMISATION

Stratification for randomisation will be by country. Within country, stratification will be by whether or not the patient was identified as eligible through the availability of an HIV antibody negative test (either within 6 months of testing HIV antibody positive or with PCR positivity).

10.2 SAMPLE SIZE

The primary endpoint is time to CD4 count decline to<350 cells/μl after 3 months from study entry on 2 occasions, within 4 weeks or to initiation of late treatment. The hypothesis to be tested is that the three groups have the same distribution of time to CD4 <350 cells/μl against the alternative that the progression rates are different. (This will be tested using a Log-rank test on 2 degrees of freedom). Data from CASCADE[55] (4000 seroconverters in Europe) gives cumulative probability of CD4 decline to <350 cells/μl by one, two and three years following seroconversion as 24%, 40% and 52% respectively in untreated patients. Applying these rates in arm C (no ART) and making the following further assumptions:

1. Total trial time is 5 years with recruitment completed in the first 18 months (i.e. average follow-up 4.25 years, minimum 3.5 years)

2. 10% lost to follow-up at the end of 5 years.

Based upon the above assumptions, a total of 360 patients (120 per group) will be required to detect (with 90% power) a relative reduction in the risk of progression to the primary endpoint of 50% and 25% in arms A (48 weeks) and B (12 weeks) respectively, relative to the no treatment arm, with significance level 5%.

For the immune function endpoint, the three groups will be compared in terms of the proportion with HIV specific CD4 response to HIV derived peptides at week 60. The expected rate in the untreated group is not known (no data) but this rate may arbitrarily be assumed to be a 10% rate in the untreated group, based on studies of immune function in long-term non-progressors. Data from the pilot study[51] suggest a 50% response rate at 6 months in treated patients. If all patients were tested, then we will be able to detect a difference between the groups with 92% power if the response rate in those treated for 48 weeks is 30% (somewhere between 10 and 30% in those treated for 12 weeks) assuming a 10% assay failure or loss to follow-up at one year. If immunology is performed in only half the patients (60 per group) then we will detect a difference with 93% power if the true

response rate in the 48-week treated arm is 40%. The same difference will be detected but with 80% power if 135 (45 per group) are part of the immunology subset.

10.3 MONITORING AND INTERIM ANALYSES

The trial Data and Safety Monitoring Committee (DSMC) will monitor the trial in all its aspects. It will consider the findings from other relevant studies. It will review unblinded data every six months, in strict confidence, on the outcome measures by treatment allocation. It may initiate interim analyses for efficacy and safety. The DSMC will advise the TSC if, in their view, the data have provided both:

- proof beyond reasonable doubt* that one of the treatment groups is better in terms of the primary outcome, and
- evidence that might be reasonably expected to materially alter the uncertainties of clinicians who are already aware of the results of other trials.

* Appropriate criteria of proof beyond reasonable doubt cannot be specified precisely but, for example, a difference of at least three standard deviations in an interim analysis of a major endpoint may be needed to justify closing or modifying such a study prematurely. If this criterion were adopted it would have the practical advantage that the exact number of interim analyses would be of little importance and so no fixed schedule is proposed.

10.4 ANALYSIS PLAN

Baseline characteristics (age, sex, likely mode of infection, estimated duration of infection, CD4 count, HIV-1 RNA) of each group will be summarised with descriptive statistics.

Efficacy data will be analysed on an intent-to-treat basis and will be performed on all randomised patients. The primary outcome will be the time to CD4 count <350 cells/µl. The global hypothesis of no difference between the three arms will be evaluated using Kaplan-Meier plots and log-rank tests (with 2 degrees of freedom) stratified by site. If this was significant (at 5% level) then treatment arms A and B will be compared using a log-rank test stratified by site.

11 ETHICAL CONSIDERATIONS

Full medical confidentiality will be observed. The Declaration of Helsinki is accepted as the basis for conducting this study. Investigators and clinical centres will sign the final protocol setting out their responsibilities and agreeing to adhere to the protocol.

11.1 SUBJECT PAYMENT

Subjects will not be paid to take part in the study, but travel expenses will be reimbursed.

12 INDEMNITY

The Sponsor will be responsible for ensuring that a policy is in place to cover 'no fault compensation' for participants on the trial who suffer an adverse event that is attributable to participation in the trial but which is not related to product or due to negligent harm caused by the clinical staff.

13 TRIAL COMMITTEES

13.1 TRIAL MANAGEMENT GROUP (TMG)

This group will oversee the day-to-day running of the trial and the members will be primarily the clinical and data management teams. Minutes of meetings will be taken and will form the basis of the progress report to the Trial Steering Committee.

13.2 TRIAL STEERING COMMITTEE (TSC)

The supervision of the trial will be responsibility of the Trial Steering Committee (TSC). The committee will consist of one principal investigator from each country with an independent chairperson and three independent members. It will also include the Trial Statistician and representatives from the TMG, the Sponsor and the Virology and Immunology groups. Additional non-voting members will attend as appropriate to the contents of the meeting.

The trial may be terminated by this Committee for any reason, including on the recommendation of the DSMC

13.3 DATA AND SAFETY MONITORING COMMITTEE (DSMC)

An independent DSMC will be established. No member of the TSC, or any clinician (investigator) responsible for the clinical care of trial patients or representative of the pharmaceutical companies may be a member of the DSMC.

14 PUBLICATION

Preparation of a manuscript for rapid publication will be the responsibility of the TSC. High priority will be given to this and it is anticipated that a report will be completed within six months of completion of the study. Any preliminary and final publications will require approval by the TSC.

15 REFERENCES

1. Spira AI, Marx PA, Patterson BK, Mahoney J, Koup RA, Wolinsky SM, *et al*. Cellular targets of infection and route of viral dissemination after an intravaginal inoculation of simian immunodeficiency virus into rhesus macaques. *J Exp Med* 1996; **183**:215-225.
2. Chun TW, Stuyver L, Mizell SB, Ehler LA, Mican JA, Baseler M, *et al*. Presence of an inducible HIV-1 latent reservoir during highly active antiretroviral therapy. *Proc Natl Acad Sci U S A* 1997; **94**:13193-13197.
3. Chun TW, Carruth L, Finzi D, Shen X, DiGiuseppe JA, Taylor H, *et al*. Quantification of latent tissue reservoirs and total body viral load in HIV-1 infection [see comments]. *Nature* 1997; **387**:183-188.
4. Pedersen C. Infection with human immunodeficiency virus type-1. Seroconversion chronic infection and the development of AIDS. *Dan Med Bull* 1994; **41**:12-22.
5. Little SJ, McLean AR, Spina CA, Richman DD, Havlir DV. Viral dynamics of acute HIV-1 infection. *J Exp Med* 1999; **190**:841-850.
6. Pedersen C, Katzenstein T, Nielsen C, Lundgren JD, Gerstoft J. Prognostic value of serum HIV-RNA levels at virologic steady state after seroconversion: relation to CD4 cell count and clinical course of primary infection. *J Acquir Immune Defic Syndr Hum Retrovirol* 1997; **16**:93-99.
7. Mellors JW, Rinaldo CRJ, Gupta P, White RM, Todd JA, Kingsley LA. Prognosis in HIV-1 infection predicted by the quantity of virus in plasma [see comments] [published erratum appears in Science 1997 Jan 3;275(5296):14]. *Science* 1996; **272**:1167-1170.
8. Wilson JD, Ogg GS, Allen RL, Davis C, Shaunak S, Downie J, *et al*. Direct visualization of HIV-1-specific cytotoxic T lymphocytes during primary infection. *AIDS* 2000; **14**:225-233.
9. McMichael AJ, Ogg G, Wilson J, Callan M, Hambleton S, Appay V, *et al*. Memory CD8+ T cells in HIV infection. *Philos Trans R Soc Lond B Biol Sci* 2000; **355**:363-367.
10. Rowland-Jones S, Dong T, Krausa P, Sutton J, Newell H, Ariyoshi K, *et al*. The role of cytotoxic T-cells in HIV infection. *Dev Biol Stand* 1998; **92**:209-214.
11. Koup RA, Safrit JT, Cao Y, Andrews CA, McLeod G, Borkowsky W, *et al*. Temporal association of cellular immune responses with the initial control of viremia in primary human immunodeficiency virus type 1 syndrome. *J Virol* 1994; **68**:4650-4655.
12. Kalams SA, Buchbinder SP, Rosenberg ES, Billingsley JM, Colbert DS, Jones NG, *et al*. Association between virus-specific cytotoxic T-lymphocyte and helper responses in human immunodeficiency virus type 1 infection. *J Virol* 1999; **73**:6715-6720.
13. Ogg GS, Jin X, Bonhoeffer S, Dunbar PR, Nowak MA, Monard S, *et al*. Quantitation of HIV-1-specific cytotoxic T lymphocytes and plasma load of viral RNA. *Science* 1998; **279**:2103-2106.
14. Moss PA, Rowland-Jones SL, Frodsham PM, McAdam S, Giangrande P, McMichael AJ, *et al*. Persistent high frequency of human immunodeficiency virus-specific cytotoxic T cells in peripheral blood of infected donors. *Proc Natl Acad Sci U S A* 1995; **92**:5773-5777.

15. Borrow P, Lewicki H, Wei X, Horwitz MS, Peffer N, Meyers H, *et al*. Antiviral pressure exerted by HIV-1-specific cytotoxic T lymphocytes (CTLs) during primary infection demonstrated by rapid selection of CTL escape virus [see comments]. *Nat Med* 1997; **3**: 205-211.

16. Borrow P, Lewicki H, Hahn BH, Shaw GM, Oldstone MB. Virus-specific CD8+ cytotoxic T-lymphocyte activity associated with control of viremia in primary human immunodeficiency virus type 1 infection. *J Virol* 1994; **68**:6103-6110.

17. Musey L, Hughes J, Schacker T, Shea T, Corey L, McElrath MJ. Cytotoxic-T-cell responses, viral load, and disease progression in early human immunodeficiency virus type 1 infection [see comments]. *N Engl J Med* 1997; **337**:1267-1274.

18. Pitcher CJ, Quittner C, Peterson DM, Connors M, Koup RA, Maino VC, *et al*. HIV-1-specific CD4+ T cells are detectable in most individuals with active HIV-1 infection, but decline with prolonged viral suppression [see comments]. *Nat Med* 1999; **5**:518-525.

19. Rosenberg ES, Billingsley JM, Caliendo AM, Boswell SL, Sax PE, Kalams SA, *et al*. Vigorous HIV-1-specific CD4+ T cell responses associated with control of viremia [see comments]. *Science* 1997; **278**:1447-1450.

20. Gotch FM, Koup RA, Safrit JT. New observations on cellular immune responses to HIV and T-cell epitopes [see comments]. *AIDS* 1997; **11 Suppl A**:S99-107.

21. Carotenuto P, Looij D, Keldermans L, de Wolf F, Goudsmit J. Neutralizing antibodies are positively associated with CD4+ T-cell counts and T-cell function in long-term AIDS-free infection. *AIDS* 1998; **12**:1591-1600.

22. Kalams SA, Walker BD. The critical need for CD4 help in maintaining effective cytotoxic T lymphocyte responses [comment]. *J Exp Med* 1998; **188**:2199-2204.

23. Cardin RD, Brooks JW, Sarawar SR, Doherty PC. Progressive loss of CD8+ T cell-mediated control of a gamma-herpesvirus in the absence of CD4+ T cells. *J Exp Med* 1996; **184**:863-871.

24. Hasenkrug KJ, Brooks DM, Dittmer U. Critical role for CD4(+) T cells in controlling retrovirus replication & spread in persistently infected mice. *J Virol* 1998; **72**:6559-656

25. Safrit JT, Koup RA. The immunology of primary HIV infection: which immune responses control HIV replication? *Curr Opin Immunol* 1995; **7**:456-461.

26. Clerici M, Via CS, Lucey DR, Roilides E, Pizzo PA, Shearer GM. Functional dichotomy of CD4+ T helper lymphocytes in asymptomatic human immunodeficiency virus infection. *Eur J Immunol*. 1991;**21**:665-70.

27. Autran B, Carcelain G, Li TS, Blanc C, Mathez D, Tubiana R, *et al*. Positive effects of combined antiretroviral therapy on CD4+ T cell homeostasis and function in advanced HIV disease [see comments]. *Science* 1997; **277**:112-116.

28. Oxenius A, Price DA, Easterbrook PJ, O'Callaghan CA, Kelleher AD, Whelan JA, *et al*. Early highly active antiretroviral therapy for acute HIV-1 infection preserves immune function of CD8+ and CD4+ T lymphocytes. *Proc Natl Acad Sci U S A* 2000; **97**:3382-3387.

29. Rinaldo C, Huang XL, Fan ZF, *et al*. High levels of anti-human immunodeficiency virus type 1 (HIV-1) memory cytotoxic T-lymphocyte activity and low viral load are associated with lack of disease in HIV-1 infected long term non-progressors. *J Virol* 1995; **69**: 5838-5842.

30. Pontesilli O, Klein MR, Kerkhof-Garde SR, Pakker NG, de Wolf F, Schuitemaker H, *et al*. Longitudinal analysis of human immunodeficiency virus type 1-specific cytotoxic T lymphocyte responses: a predominant gag-specific response is associated with nonprogressive infection. *J Infect Dis* 1998; **178**:1008-1018.

31. Altfeld, M., Rosenberg, E., Shankarappa, R *et al*. Cellular immune responses and viral diversity in individuals treated during acute and early HIV-1 infection. *J Exp Med* 2001;**193**: 169-180.

32. Rosenberg E, Altfeld M, Poon S, *et al*. Immune control of HIV-1 after early treatment for acute infection. *Nature* 2000; **407**: 523-526.

33. Bart PA, Rizzardi GP, Tambussi G, *et al*. Immunological and virological responses in HIV-1 infected adults at early stage of established infection treated with highly active antiretroviral therapy. *AIDS* 2000; **14**: 1887-1897.

34. Kaufman GR, Zaunders JJ, Cunningham P, *et al*. Rapid restoration of CD4 T-cell subsets in subjects receiving antiretroviral therapy during primary HIV infection. *AIDS* 2000; **14**: 2643-2651.

35. Carcelain G, Blanc C, Leibowitch J, Mariot P, Mathez D, Schneider V, *et al*. T cell changes after combined nucleoside analogue therapy in HIV primary infection. *AIDS* 1999; **13**:1077-1081.

36. Hel Z, Venzon D, Poudyal M, Tsai WP, Giuliani L, Woodward R, *et al*. Viremia control following antiretroviral treatment and therapeutic immunization during primary SIV251 infection of macaques [In Process Citation]. *Nat Med* 2000; **6**:1140-1146.

37. Lifson JD, Rossio JL, Arnout R, *et al*. Containment of simian immunodeficiency virus infection: cellular immune responses and protection from rechallenge following transient post inoculation antiretroviral treatment. *J Virol* 2000; **74**: 2584-2593.

38. Malhotra U, Berrey MM, Huang Y, *et al*. Effect of combination antiretroviral therapy on T-cell immunity in acute Human immunodeficiency virus infection. *J Infect Dis* 2000; **181**: 121-131.

39. Hoen B, Dumon B, Harzic M, *et al*. Highly active antiretroviral treatment initiated in the course of symptomatic primary HIV-1 infection: results of the ANRS 053 trial. *J Infect Dis* 1999; **180**: 1342-6.

40. Markowitz M, Vesanen M, Tenner-Racz K, *et al.* The effect of commencing combination antiretroviral therapy soon after human immunodeficiency virus type 1 infection on viral replication and anti-viral responses. *J Infect Dis* 1999; **179**: 527-537.

41. Miller J, Carr A, Smith D, Emery S, *et al*. Lipodystrophy following antiretroviral therapy of primary HIV infection. *AIDS* 2000; **14**: 2406-2407.

42. Klein MR, van Baalen CA, Holwerda AM, Kerkhof GS, Bende RJ, Keet IP, *et al*. Kinetics of Gag-specific cytotoxic T lymphocyte responses during the clinical course of HIV-1 infection: a longitudinal analysis of rapid progressors and long-term asymptomatics. *J Exp Med* 1995; **181**:1365-1372.

43. Ogg GS, Jin X, Bonhoeffer S, Moss P, Nowak MA, Monard S, *et al*. Decay kinetics of human immunodeficiency virus-specific effector cytotoxic T lymphocytes after combination antiretroviral therapy. *J Virol* 1999; **73**:797-800.

44. Pontesilli O, Klein MR, Kerkhof-Garde SR, Pakker NG, de Wolf F, Schuitemaker H, *et al*. Kinetics of immune functions and virus replication during HIV-1 infection. *Immunol Lett* 1997; **57**:125-130.

45. Kaufmann D, Bailey P, Brander C, Truong H, *et al*. Kinetics of HIV-specific CD4+ T-cell responses in subjects treated with HAART during Primary HIV infection. 10th Conference on Retroviruses and Opportunistic Infections, 10th- 14th February 2003, Boston [151].

46. Kaufmann GR, Zaunders JJ, Cunningham P, *et al*. Rapid restoration of CD4 T-cell subsets in subjects receiving antiretroviral therapy during primary HIV infection. *AIDS* 2000; **14**: 2643-51.

47. Yerly S, Kaiser L, Perneger TV, *et al*. Time of initiation of antiretroviral therapy; impact on HIV-1 viraemia. *AIDS* 2000; **14**: 243-9.

48. Stanford SA, Ong JC, Martinez-Marino B, et al. Reduction in non-cytotoxic anti-HIV activity in individuals receiving HAART during primary HIV infection. *PNAS* 2001; **98**: 597-602.

49. Orenstein JM, Bhat N, Yoder C, Fox C, Polis MA, Metcalf JA, *et al*. Rapid activation of lymph nodes and mononuclear cell HIV expression upon interrupting highly active antiretroviral therapy in patients after prolonged viral suppression [In Process Citation]. *AIDS* 2000; **14**:1709-1715.

50. Davey RTJ, Bhat N, Yoder C, Chun TW, Metcalf JA, Dewar R, *et al*. HIV-1 and T cell dynamics after interruption of highly active antiretroviral therapy (HAART) in patients with a history of sustained viral suppression. *Proc Natl Acad Sci U S A* 1999; **96**: 15109-15114.

51. Fidler S, Oxenius A, Brady M, *et al*. Virological and immunological effects of short course antiretroviral therapy in primary HIV infection. *AIDS* 2002; **16**: 2049- 2054.

52. Phillips AN, Staszewski S, Weber R, Kirk O, Francioli P, Miller V, Vernazza P, Lundgren JD, Ledergerber B; Swiss HIV Cohort Study; Frankfurt HIV Clinic Cohort; EuroSIDA Study Group. HIV viral load response to antiretroviral therapy according to the baseline CD4 cell count and viral load. *JAMA* 2001;**286**:2560-7.

53. Lacabaratz-Porret C, Urrutia A, Doisne JM, Goujard C, Deveau C, Dalod M, Meyer L, Rouzioux C, Delfraissy JF, Venet A, Sinet M **.**Impact of antiretroviral therapy and changes in virus load on human immunodeficiency virus (HIV)-specific T cell responses in primary HIV infection. *J Infect Dis*. 2003;**187**:748-57.

54. Mackie N, Fidler S, Tamm N, Clarke JR, Back D, Weber JN, Taylor GP. Clinical implications of stopping nevirapine-based antiretroviral therapy: relative pharmacokinetics and avoidance of drug resistance. *HIV Medicine* (in press 2004).

55. CASCADE Collaboration. Changes in the uptake of antiretroviral therapy and survival in persons with known duration of HIV infection in Europe. *HIV Medicine* 2000;1:224-31.

56. Honkoop, P, de Man RA, Niesters, HG, Zondervan, PE, Schalm, SW. Acute exacerbation of chronic hepatitis B infection after withdrawal of lamivudine therapy. *Hepatology* 2000 3 635-9.

Appendix 1

ANTIRETROVIRAL DRUG INFORMATION
Recommended doses and common side effects of treatments

Nucleoside Reverse Transcriptase Inhibitors (NRTIs)

Generic	Brand	Dose	Comments and Common Side Effects
Abacavir (ABC)	*Ziagen*	300 mg 2x/d	About 4% hypersensitivity reaction: fever, malaise, possible rash, GI, respiratory. Resolves within 2 days after discontinuation. DO NOT RECHALLENGE. Also: rash alone without hypersensitivity.
Didanosine (ddI)	*Videx EC*	400-mg capsule 1x/d on empty stomach (>60 kg body weight)	Peripheral neuropathy in 15%, rare pancreatitis; avoid alcohol. OK to take *Videx EC* at same time as other antiretrovirals that can be taken on an empty stomach. Chewable tablet formulation has additional restrictions.
Lamivudine (3TC)	*Epivir*	150 mg 2x/d	Generally well tolerated. Active against HBV.
Stavudine (d4T)	*Zerit*	40 mg 2x/d (>60 kg body weight)	Peripheral neuropathy (1%-4% in early studies; 24% in expanded access patients with CD4+ counts <50).
Zalcitabine (ddC)	*Hivid*	0.375-0.75 mg 3x/d	Peripheral neuropathy in 17%-31% of trial participants; oral ulcers. Used rarely due to toxicity, inconvenient dosing, and questions regarding efficacy.
Zidovudine (ZDV, AZT)	*Retrovir*	300 mg 2x/d	Initial nausea, headache, fatigue, anaemia, neutropenia, neuropathy, myopathy.
ZDV + 3TC	*Combivir*	1 tablet 2x/d	Combination tablet contains 300mg ZDV/150mg 3TC
ZDV + 3TC + ABC	*Trizivir*	1 tablet 2x/d	Combination tablet containing 300 mg of ZDV, 150 mg of 3TC, and 300 mg of abacavir.
Tenofovir	*Viread*	300 mg 1x/d with food	Generally well tolerated. Significant interaction with ddI (see below). Active against HBV.

Protease Inhibitors (PIs)

Generic	Brand	Dose	Comments and Common Side Effects
Amprenavir	*Agenerase*	1200 mg (8 cap) 2x/d, or 600 mg with RTV 100 mg 2x/d, or 1200 mg with RTV 200 mg 1x/d *	Rash (20%), diarrhoea, nausea
Indinavir	*Crixivan*	800 mg (2 cap) every 8 hours on empty stomach or with snack containing <2 g of fat *	Kidney stones in 6%-8%: good hydration essential. Occasional nausea and GI upset. Store in original container -contains desiccant without this, IDV is stable for about 3 days.
Lopinavir/ Ritonavir	*Kaletra*	Co formulated lopinavir 400 mg + ritonavir 100 mg (3 cap) 2x/d with food	GI side effects common but mild. Hyperlipidemia.
Nelfinavir	*Viracept*	1250 mg (5 tab) 2x/d or 750 mg (3 tab) 3x/d with food	Diarrhoea common; occasional nausea

Ritonavir	*Norvir*	600 mg (6 cap) 2x/d; start with 300 mg 2x/d and increase to full dose over 14 days	Nausea, diarrhoea, numb lips; occasional hepatitis; hyperlipidaemia. Store capsules in refrigerator. Stable at room temperature for up to 1 month. Used at lower dosages as pharmacokinetic enhancer of other PIs.
Saquinavir soft gel cap	*Fortovase*	1600 mg (8 cap) 2x/d or 1200 mg (6 cap) 3x/d with fat-containing food (>28 g) *	Soft gel formulation with improved absorption. Long-term storage in refrigerator. Stable at room temperature for 3 months.
Saquinavir hard gel cap	*Invirase*	Used in combination with ritonavir	Hard gel formulation with poor absorption. Bioequivalent to *Fortovase* when combined with RTV. Smaller tablet size and easier storage than *Fortovase*.

* Frequently dosed with ritonavir to simplify administration and raise drug levels. See Drug-Drug Combinations section for details.

Non-nucleoside Reverse Transcriptase Inhibitors (NNRTIs)

Generic	Brand	Dose	Comments and Common Side Effects
Efavirenz	*Sustiva*	600 mg (1 tab) 1x/d initially at bedtime	Initial dizziness, insomnia, transient rash, P450 3A4 inducer; avoid clarithromycin coadministration.
Nevirapine	*Viramune*	200 mg (1 tab) 1x/d for 2 weeks, then 200 mg 2x/d or 400 mg 1x/d	Transient rash, hepatitis; thus, monitor LFTs. P450 3A4 inducer. Once-daily dosing recommendation based on limited clinical data.

Drug-Drug Combinations

This table gives an overview of current knowledge of drug-drug combinations. Except for Tenofovir/ ddI interaction, the NRTIs are not listed but may require dose adjustments when combined (please refer if unsure). PIs and NNRTIs tend to have complex metabolism and in combinations affect each other's drug levels and potency. The knowledge on these combinations is still evolving, and few formal dose modification recommendations are available. Caution and close monitoring are advised. Treating physicians should verify all information with an AIDS specialist and check any dose adjustments with a pharmacist.

Abbreviations

RTV	Ritonavir
IDV	Indinavir
NFV	Nelfinavir
APV	Amprenavir
LPV	Lopinavir
SQV	Saquinavir (Fortovase, Invirase)

NVP	Nevirapine
DLV	Delavirdine
EFV	Efavirenz
ADV	Adefovir
TNV	Tenofovir

	APV	IDV	LPV/RTV	NFV	RTV	SQV	DLV	EFV
**NVP**	No data	↓IDV	↓LPV	No significant interaction	No significant interaction	↓SQV	No data	↓EFV
**EFV**	↓APV	↓IDV	↓LPV	No significant interaction	Modest ↑ in both	↓SQV level; do not combine	No data	

<table>
<tr><td>DLV</td><td>APV</td><td>↑IDV</td><td>No data</td><td>↑NFV</td><td>↑RTV</td><td>↑SQV</td><td></td><td></td></tr>
<tr><td>SQV</td><td>↓APV;
↓SQV</td><td>Antagonistic in vitro (in one lab)</td><td>↑SQV-S</td><td>↑SQV-S</td><td>↑SQV</td><td></td><td></td><td></td></tr>
<tr><td>RTV</td><td>↑APV;
↓RTV</td><td>↑IDV</td><td>↓RTV</td><td>↑NFV</td><td></td><td></td><td></td><td></td></tr>
<tr><td>NFV</td><td>↑APV</td><td>↑IDV</td><td>↑NFV</td><td></td><td colspan="4" rowspan="3">Tenofovir/didanosine interaction
Tenofovir increases ddI (EC) Cmax by +49% and AUC by +46% when doses separated by 2 hours. When co administered with food the effect is enhanced (Cmax +64%; AUC +60%). ddI dose should be reduced to 250mg.</td></tr>
<tr><td>LPV/RTV</td><td>↓APV;
↓LPV</td><td>↑IDV</td><td></td><td></td></tr>
<tr><td>IDV</td><td>↑APV;
↓IDV</td><td></td><td></td><td></td></tr>
<tr><td colspan="9">Contraindicated Combinations
•ZDV + d4T combination is antagonistic in vivo
•ddI and ddC should not be combined due to increased risk of peripheral neuropathy
• IDV + SQV combination is antagonistic in vitro and in practice extremely difficult to dose
• d4T + ddI is contraindicated based on toxicity</td></tr>
</table>

Appendix 2

SAMPLE PATIENT INFORMATION SHEET AND CONSENT FORMS
[Each country to use its own translated Patient Information Sheet and Consent Forms according to local regulatory requirements, on local headed paper]

Patient Information

The SPARTAC TRIAL
A multi-centre randomised trial of therapeutic intervention at primary HIV infection

You are being invited to take part in a research study. Before you decide, it is important for you to understand why the research is being done and what it will involve. Please take time to read the following information carefully and discuss it with others if you wish. Ask us about anything that is not clear or if you would like more information. Thank you for reading this.

Why is this study being done?
HIV infects and destroys the cells of the immune system, which normally protect against infections. Progressively the immune system may become too weak to offer any protection. This process is slow and, on average, people remain well for 10 years, although this varies greatly between individuals. Detailed studies of the immune system have shown that from the earliest stage of HIV infection, termed 'primary infection' your immune system is weakening.

The majority of people with HIV do not take anti-HIV drugs when first infected generally because they don't know that they've recently been infected but also because, once started, therapy is likely to be life-long and it is best to try to minimise time spent on therapy. There have been several recent, small studies looking at treatment in primary infection. Although some have shown that the immune system may benefit from early treatment, they have not been able to show whether taking antiretroviral treatment early has any benefit in delaying damage to the immune system. Clearly, unless it can show that, there may be no gain in taking these therapies early in infection. At present we do not know whether treating an HIV infected person immediately following infection is more beneficial to delaying treatment and this is why this large, long-term clinical trial is being conducted.

We would, therefore, like to compare 3 groups of patients who've been identified during primary infection; a group who take a short course of treatment (12 weeks), a group who take a longer course (48 weeks) and a group who will have no treatment. You will be randomised to one of these groups (like the toss of a coin) and have the same chance (a third) of being put into any one of them. You will not have a choice which group to go into.

Why am I being asked?
Because you have contracted HIV during the last 6 months and, therefore, are in a minority of people whose time of infection is known.

Do I have to take part?
No you don't have to as participation is entirely voluntary. It is up to you to decide whether or not to take part. If you do decide to take part you will be given this information sheet to keep and be asked to sign a consent form. If you decide to take part you are still free to withdraw at any time and without giving a reason.

It is important you feel that you have some understanding of your HIV diagnosis and what this means before you consider taking part in this study. You cannot choose which arm of the study you are allocated to and so you must be happy to be put into any of the groups. If you decide not to take part in this study this will in no way affect your clinical care and you can discuss your future care with your clinic doctor.

What will happen to me if I take part?
You will be involved in the study for 3.5 to 5 years (depending on when you join). After you have signed the consent form you will have a medical check up and be asked questions about your health and any medicines you are taking.

You will then have about 40 mls (3 tablespoons) of blood taken for routine tests plus those for viral hepatitis and levels of cholesterol and triglycerides (fats in the blood). We will repeat the routine screening tests for both Hepatitis B and C viruses annually. As well, your blood will be tested to see if your HIV is resistant to any HIV drugs. The results of these tests will be provided to you when they are available.

Women who could become pregnant will have a urine or blood test to exclude pregnancy.

If this is the first opportunity you've had to find out about this study, we need you to come back in the next 2 weeks when you will be randomised into one of the 3 arms of the study. The number of patients in each group will be the same so you have a 1 in 3 chance of being put into any one group.

If you are randomised to one of the treatment arms the medication you will most likely be offered is Combivir and Kaletra. Combivir is one tablet containing 2 drugs (zidovudine/lamivudine), which is taken twice a day; Kaletra is three capsules, containing lopinavir with a small boosting dose of ritonavir, also taken twice a day. One of the doctors or nurses will help you decide when and how to take the medication before you start. If for any reason this combination is not suitable your doctor can prescribe a different combination.

You will then be asked to attend the clinic regularly. This will be more frequent in the first year and then once every 3 months or so until the end of the study. At each visit a general medical check will be performed to make sure you are well and are not experiencing side effects from the drugs.

Apart from bloods for your routine care, we would also like some of this blood for research purposes to help us understand exactly how your immune system is fighting HIV. We will also determine your HLA (tissue) type and will test for the presence of any viral drug resistance. Genetic tests will also be done on a stored coded (i.e. without personal identifiers) sample of your DNA.

If you feel unwell or are worried about your health we will be happy to see you at other times.

What is a genetic test?
A genetic test is one carried out on a sample of your DNA. This is because, to an extent, how your body responds to an infection is affected by your genetic make-up. We inherit genes (genetic material) from our parents and the unique combination of these genes controls many body functions. There are certain known genetic factors that influence how your immune response develops but others, not yet known, may also play an important role. The tests to identify any such genes are done on samples of blood and involve your unique set of DNA being extracted.

DNA will be extracted at a future date from one of the blood samples being taken from you. Your specific consent is needed for this part of the study and does not affect you taking part in the main SPARTAC study. However, being able to match up the results of genetic tests with how your immune system is coping with this new infection and monitoring this over time is very valuable and has never been done before.

Because little is known about the significance of any findings from your stored DNA, and because these tests will be performed at some, as yet undetermined date in the future, you will not be given results from findings related to this part of the study. However, you can be assured that any

findings relating to the presence of new genetic changes that may affect disease outcome will be made available to all study participants.

Will the study benefit me?
If you take part in the study, there may be a direct benefit to you but no guarantee can be made. You will receive the results of some tests that are not part of your routine care. It is possible that you may receive no benefit from being in this study. The information we get from this study may help us treat patients with HIV infection better in the future.

What are the risks?
There is a risk that you may feel dizzy or light headed after having blood taken and, if you are to be given anti-HIV drugs, you will also have the inconvenience of having to take regular medication for either 12 or 48 weeks.

If you are randomised to take drug therapy you may experience some side effects. Most drugs are capable of producing side effects, HIV drugs are no different in this way. However, some HIV drugs are more likely to produce side effects than others. The combination your doctor chooses for you will be based on the most powerful and yet best tolerated drugs we have to treat HIV. The main side effects we would expect using these drugs are:

- Feeling nauseous or vomiting
- Skin rashes
- Increase in blood fats (lipids)
- Changes in your liver function or an inflamed liver
- Diarrhoea
- Headaches
- Anaemia (low blood count)
- Dizziness

Many of these side effects pass without having to stop the treatment and medication to prevent/control them can be given. If any of these side effects were unacceptable we would consider changing the medication for something that suits you better.

We will check to see if the HIV you contracted has any evidence of drug resistance – this is where the virus changes its structure so that the drugs no longer work. If you take the antiretrovirals regularly and without missing any doses there is no reason that this could affect your future drug treatment. But if you miss doses regularly the virus could become resistant to the drugs and no longer be controlled by the treatment combination.

What if something goes wrong?
If you are harmed as a result of participation in this study and it is not due to negligence Imperial College (London) accepts liability attached to its management of the trial and would sympathetically consider any claim for compensation. Your hospital is responsible for your care during the study and all hospitals have procedures for dealing with complaints or any concerns about any aspect of the way you have been approached or treated during the course of the study.

What happens if I change my mind?
This study is completely voluntary and you can withdraw at any time without giving a reason. Withdrawal from the study will NOT affect your future medical care at the centre in any way.

What happens if I get ill?
Most people in your early stage of HIV infection do not take drug treatment. If for some reason you should fall ill, you will be treated in exactly the same way as you would have been had you not taken part in this study.

What happens if new information becomes available?
Sometimes during the course of a study, new information becomes available about the strategy that is being tested. If this happens, your doctor will tell you about it and discuss its implications with you. Taking part in this study will not disqualify you from having access to new treatments.

Will my taking part in this study be kept confidential?
All information that is collected about you during the course of the study will be kept strictly confidential. When you consent to take part in this study you must agree to allow authorised staff from the Medical Research Council (MRC) Clinical Trials Unit or a regulatory authority to inspect your medical records to monitor the study. In no circumstances will your name, address or telephone number be disclosed outside the clinic.

We would like you to let us tell your GP that you have entered this study. However, if you decline to do so this will be respected and you can still join the study.

Some blood that is being collected from you will be frozen and put in storage and may only be used during the trial for tests related to HIV. These tests would only look at characteristics of HIV, such as resistance to HIV drugs, which could help explain the results of the trial and contribute to our knowledge of HIV infection. By signing the consent form you are authorising the use of this blood for these future tests. This blood will be stored coded, i.e. without any personal identifiers, only your trial number and date of birth. Some of the blood will be stored at the Peter Medawar Centre in Oxford and the rest at Imperial College in London, and it will all be stored for up to 5 years, which is the lifetime of this study, after which time it will be discarded. Application for use of these specimens by clinical researchers can only be done through the Trial Steering Committee.

DNA samples for genetic testing will only be available to this trial and used only for the purposes of this research. Additionally an application for the use of these DNA samples will have to be made to the London Multi-Centre Research Ethics Committee.

What will happen to the results of the research study?
After the study is completed there will be an analysis. This usually takes 3-6 months and after this you will be told the results of the study. The results of the study will be written up and submitted to a medical journal. They may also be presented at scientific conferences.

Will I be paid for taking part in the study?
You will not be paid to take part in the study but your travel expenses will be reimbursed.

Who is organising and funding the research?
The research is being organised by the UK Medical Research Council Clinical Trials Unit and is funded by a Wellcome Trust Grant given to Imperial College.

Who has reviewed this study?
The study has been reviewed by a Multi-centre Research Ethics Committee (MREC) and also by the local committee for this hospital.

Contact for further information
If you would like to talk to someone about this study please contact:

--
Names of Study Doctor and Nurse plus contact numbers to be added here.

Thank you for considering to take part in this study.

To be printed on local headed paper

Consent Form

The SPARTAC TRIAL: A Multi-centre, randomised trial of therapeutic intervention at primary HIV infection (PHI)

Please initial box if you agree

I have read the Spartac (*insert version*) Patient Information sheet ☐

I have had an opportunity to ask questions and discuss this trial ☐

I have received satisfactory answers to all of my questions ☐

I have received enough information about the trial ☐

I agree for appropriate members of Medical Research Council Clinical Trials Unit and the regulatory authorities having access to your medical records ☐

Which study doctor or nurse have you spoken to about this trial?
- PLEASE PRINT HIS/HER NAME...

I understand that I am are free to withdraw from the trial:
- **at any time without having to give a reason**
- **and without affecting future medical care** ☐

I understand that coded information about me will be held electronically and will be seen by all investigators working on the trial ☐

I agree to blood being taken for tissue-typing ☐

I agree that my stored blood specimens from this trial can be tested during the lifetime of this study for tests relating to the trial ☐

I agree to my GP being informed ☐

I agree to take part in this trial ☐

____

Name of patient Date Signature

____

Name of researcher Date Signature

IMPORTANT: One signed original to be given to patient (with a copy of the Patient Information Sheet)
One signed original to be kept on file by the researcher
One signed photocopy to be kept in the hospital notes

To be printed on local headed paper

Genetic Tests Consent Form

The SPARTAC TRIAL: A Multi-centre, randomised trial of therapeutic intervention at primary HIV infection (PHI)

Please initial box if you agree

Statement	Initials
I have read the Spartac (*insert version*) Patient Information sheet	
I have had an opportunity to ask questions and discuss this trial	
I have received satisfactory answers to all of my questions	
I understand I will not receive the results of these genetic tests	
I understand that the samples taken will be stored and may be used again within the lifetime of the study for similar tests	
I understand that I can withdraw from this part of SPARTAC at any time and that the samples will then be destroyed	
I agree for blood samples to be taken for genetic testing	

____

Name of patient Date Signature

____

Name of researcher Date Signature

IMPORTANT: One signed original to be given to patient (with a copy of the Patient Information Sheet)
One signed original to be kept on file by the researcher
One signed photocopy to be kept in the hospital notes

Appendix 3

SAMPLE GP LETTER
[Each country to use its own GP letter according to local regulatory requirements, on local headed paper]

The SPARTAC study
A Multi-Centre Randomised Trial of Therapeutic Intervention at Primary HIV Infection (PHI)

Dear Dr ....,

Your patient, ..., has consented to participate in this randomised clinical trial.

The trial is an open, randomised trial comparing a long course (48 weeks) of antiretroviral therapy, a short course (12 weeks) of therapy with no therapy.

The primary aim of the study is to determine the effect of anti-retroviral therapy of limited duration in PHI on CD4 cell decline and therefore delaying the time to starting long-term HIV treatment.

Your patient was invited to participate in the trial after recently contracting HIV and was referred to me as the study physician for this clinical trial.
Your patient has been randomised to the……………………………….. treatment arm and will be followed-up in the trial for up to 5 years.

The treatment your patient has been prescribed is:

1……………………………………………………………………………….…

2……………………………………………………………………………..……

3………………………………………………………………………………..

During the course of the study, I will keep you informed of the patient's progress. If you have any questions regarding the trial or any concerns regarding this patient's inclusion in this trial, please do not hesitate to contact me.

Yours sincerely (etc)

Appendix 4

DEFINITIONS AND CRITERIA FOR AIDS EVENTS

Based on the 1993 US Centres for Disease Control and Prevention definitions (MMWR 1992; 41 [No RR-17]: 1-19).

CATEGORY C, AIDS with presumptive and definitive criteria.

	PRESUMPTIVE CRITERIA	DEFINITIVE CRITERIA
CONSTITUTIONAL DISEASE		
HIV Wasting syndrome	Unexplained, involuntary weight loss >10% from baseline (week 0) PLUS persistent diarrhoea with 2 or more liquid stools/day > 1 month OR chronic weakness OR persistent fever > 1 month. Should exclude other causes such as cancer, TB, MAI, cryptosporidiosis or other specific enteritis	none
INFECTIONS		
Candidiasis of bronchi, trachea or lungs	none	macroscopic appearance at bronchoscopy or autopsy, or histology or cytology/smear (not culture)
Oesophageal candidiasis	Recent onset of retrosternal pain on swallowing PLUS a clinical diagnosis of oral candidiasis or by cytology smear (not culture), PLUS clinical response to treatment	macroscopic appearance at endoscopy or autopsy, or histology or cytology/smear (not culture)
Coccidiodomycosis, disseminated or extrapulmonary	none	histology or cytology, culture or antigen detection from affected tissue
Cryptococcosis, meningitis or extrapulmonary	none	histology or cytology/microscopy, culture or antigen detection from affected tissue
Cryptosporidiosis	none	persistent diarrhoea > 1 month, histology or microscopy
CMV retinitis	Symptomatic or asymptomatic. Typical appearance on fundoscopy of discrete patches of retinal whitening, spreading along blood vessels, associated with vasculitis, haemorrhage and necrosis, confirmed by ophthalmologist.	none
CMV end-organ disease	none	compatible symptoms, plus histology or detection of antigen from affected tissue
CMV radiculomyelitis	Leg weakness and decreased reflexes or syndrome consistent with cord lesion presenting subacutely over days to weeks. Myelogram shows no mass lesion. CSF shows >5 WBC with >50% polymorphs and no other pathogen or persistence of symptoms after appropriate treatment for other pathogens, OR CMV shown by PCR, antigen or culture	none
CMV meningoencephalitis	Rapid (days to 1-4 weeks) syndrome with progressive delirium, cognitive impairment +/- seizures and fever (often with other CMV disease elsewhere). CT/MRI may show periventricular abnormalities with or without contrast enhancement. CSF may be normal or show evidence of CMV	none
	PRESUMPTIVE	DEFINITIVE
HSV visceral disease, e.g. bronchitis, pneumonitis,	none	symptoms, plus histology or culture or detection of antigen from affected tissue

oesophagitis		
Histoplasmosis, disseminated or extrapulmonary	none	symptoms, plus histology or culture or detection of antigen from affected tissues
Isosporiasis	none	persistent diarrhoea > 1 month, histology or microscopy
Leishmaniasis, visceral	none	symptoms, plus histology
Microsporidiosis	none	persistent diarrhoea > 1 month, histology or microscopy
MAC, and other atypical mycobacteriosis	Symptoms of fever, fatigue, anaemia or diarrhoea, plus AFBs seen in stool, blood, body fluid or tissue but not grown on culture, and no concurrent diagnosis of TB, except pulmonary	symptoms of fever, fatigue, anaemia or diarrhoea, culture from stool, blood, body fluid or tissue, except pulmonary
Tuberculosis, pulmonary	Symptoms of fever, dyspnoea, cough, weight loss or fatigue, plus AFBs seen in sputum or lavage or lung tissue but not grown in culture, plus responds to standard TB treatment	symptoms of fever, dyspnoea, cough, weight loss or fatigue, plus culture from sputum or lavage or lung tissue
Tuberculosis, extrapulmonary	Symptoms, plus AFBs seen from affected tissue or blood but not grown in culture, concurrent diagnosis of pulmonary TB or responds to standard TB treatment	symptoms, plus culture from blood or affected tissue
PCP	Recent symptoms, plus typical CXR appearance if on PCP prophylaxis or any CXR appearance if not on prophylaxis and CD4 <200, negative bronchoscopy if already treated for PCP for > 7 days or not done, no bacterial pathogens in sputum, and responds to PCP treatment	microscopy or histology
Extrapulmonary *pneumocystis*	none	symptoms plus microscopy or histology
Recurrent bacterial pneumonia	Second pneumonic episode within 1 year, new CXR appearance, symptoms and signs, diagnosed by a doctor	second pneumonic episode with 1 year, new CXR appearance, detection of bacterial pathogen
PML, Progressive multifocal leukoencephalopathy	Symptoms and brain scan consistent with PML, and no response to toxo treatment	histology
Recurrent salmonella septicaemia	none	second distinct episode, culture
Cerebral toxoplasmosis	Symptoms of focal intracranial abnormality or decreased consciousness, and brain scan consistent with lesion(s) having mass effect or enhanced by contrast, and either positive toxoplasma serology or responds to treatment clinically and by scan	histology or microscopy
Other extrapulmonary toxoplasmosis	none	symptoms plus histology or microscopy

	PRESUMPTIVE	DEFINITIVE
NEOPLASMS		
KS, Kaposi's sarcoma	Typical appearance without resolution. clinicians who have seen few cases should not make presumptive diagnoses	Histology
Primary cerebral lymphoma	Symptoms consistent with lymphoma, at least one lesion with mass effect on brain scan, no response clinically and by scan to toxoplasma treatment	
B-cell, non-Hodgkin's lymphoma	none	histology
Cervical carcinoma, invasive	none	histology, not carcinoma-in-situ
NEUROLOGICAL		
HIV encephalopathy	Cognitive or motor dysfunction interfering with usual activity, progressive over weeks or months in the absence of another condition to explain the findings, should have brain scan +/- CSF to exclude other causes. should be grade 2 or worse in at least 2 domains by NARS (see below) excluding abnormal domains at trial entry	none
OTHER		
Indeterminate intracerebral lesion(s)	Neurological illness with evidence for an intracerebral lesion(s) by brain scan where the differential diagnosis is either cerebral toxoplasmosis, PML, cerebral lymphoma or HIV encephalopathy	none

ABBREVIATED NARS (Neuropsychiatric AIDS Rating Scale) grading for <u>HIV ENCEPHALOPATHY</u>

Adapted from: Price RW, Brew BJ. The AIDS dementia complex. J Infect Dis 1988; 158 (5): 1079-83, and
Hughes CP, Berg L, Danziger WL. A new clinical scale for the staging of dementia. Brit J Psych 1982; 140: 566-92.

NARS stage	Cognitive-Behavioural Domains					
	Orientation	Memory	Motor	Behaviour	Problem solving	Activities of daily living
0.5	fully oriented	complains of memory problems	fully ambulatory slightly slowed movements	normal	has slight mental slowing	slight impairment in business dealings
1	fully oriented, may have brief periods of "spaciness	mild memory problems	balance, co-ordination and handwriting difficulties	more irritable, labile or apathetic, withdrawn	difficulty planning and completing work	can do simple daily tasks, may need prompting
2	some disorientation	memory moderately impaired, new learning impaired	ambulatory but may require walking aid	some impulsivity or agitated behaviour	severe impairment, poor social judgement, gets lost easily	needs assistance with ADLs
3	frequent disorientation	severe memory loss, only fragments of memory remain	ambulatory with assistance	may have organic psychosis	judgement very poor	cannot live independently
4	confused and disoriented	virtually no memory	bedridden	mute and unresponsive	no problem solving ability	nearly vegetative

Appendix 5

ADHERENCE QUESTIONNAIRE

Date:	Clinic no:	Initials:	Date of birth:	Trial no:

Please tell us if you have been unable to take your anti-HIV treatment as planned.
You do not have to answer if you do not want do. Complete before seeing your doctor.

A. During the past 4 days, on how many days have you missed taking all (or part) of your doses? (tick):
One day ☐ Two days ☐ Three days ☐ Four days ☐ None ☐ (If None, go to question C)

B. Write in the boxes the number of doses (or part of a dose) missed during the past 4 days. Write "0" if you did not miss a dose

	How many doses did you miss?			
**Step 1.** Write in the names of your anti-HIV drugs:	**Step 2** Yesterday	**Step 3** 2 days ago	**Step 4** 3 days ago	**Step 5** 4 days ago
	I missed doses	I missed doses	I missed doses	I missed doses
	I missed doses	I missed doses	I missed doses	I missed doses
	I missed doses	I missed doses	I missed doses	I missed doses
	I missed doses	I missed doses	I missed doses	I missed doses
	I missed doses	I missed doses	I missed doses	I missed doses

C. Which one (if any) of your anti-HIV drugs is the easiest to take? (write in here):
....

D. Which one (if any) of your anti-HIV drugs is the most difficult to take? (write in here):
....

E. Most anti-HIV drugs need to be taken on a schedule, such as "twice a day", or "three times a day" or "every 12 hours". How closely did you follow your specific schedule over the last 4 days? (tick):
Never ☐ Some of the time ☐ About half of the time ☐ Most of the time ☐ All of the time ☐

F. Do any of your anti-HIV drugs have special instructions, such as "take with food", or "on an empty stomach"?
(tick): Yes ☐ No ☐
If Yes, how often did you follow these special instructions over the last 4 days? (tick):
Never ☐ Some of the time ☐ About half of the time ☐ Most of the time ☐ All of the time ☐

G. Some people find that they forget to take their pills on the weekends. Did you miss any of your anti-HIV drugs last weekend (last Saturday or Sunday)? (tick): Yes ☐ No ☐

H. When was the last time you missed any of your anti-HIV drugs? (tick):
Within the last week ☐ 1-2 weeks ago ☐ 2-4 weeks ago ☐ 1-3 months ago ☐ More than 3 months ago ☐ Never ☐
(If you never miss your drugs do not answer the next question)

I. People may miss taking their anti-HIV drugs for many reasons. Here is a list of possible reasons. How often have you missed taking your anti-HIV drugs because you: (tick one box on each line):

	**Never**	**Rarely**	**Sometimes**	**Often**
1. Were away from home?	☐	☐	☐	☐
2. Were too busy with other things?	☐	☐	☐	☐
3. Simply forgot?	☐	☐	☐	☐
4. Had too many pills to take?	☐	☐	☐	☐
5. Wanted to avoid side effects?	☐	☐	☐	☐
6. Did not want other people to notice you taking pills?	☐	☐	☐	☐
7. Had a change in daily routine?	☐	☐	☐	☐
8. Felt like the drug was toxic or harmful?	☐	☐	☐	☐
9. Were asleep through the dose time?	☐	☐	☐	☐
10. Felt sick or ill?	☐	☐	☐	☐
11. Felt depressed or overwhelmed?	☐	☐	☐	☐
12. Had a problem taking pills at a specified time (with meals or on an empty stomach)?	☐	☐	☐	☐
13. Ran out of pills?	☐	☐	☐	☐
14. Felt good?	☐	☐	☐	☐

Appendix 6

GRADING OF ADVERSE EVENTS

ULN= upper limit of local reference range (“upper limit of normal”)

	Grade 1	Grade 2	Grade 3	Grade 4
HAEMATOLOGICAL				
Haemoglobin g/dl	9.5-10.5	8.0-9.4	6.5-7.9	<6.5
Leucopoenia 10^9/l	3.0-3.9	2.0-2.9	1.9-1.0	<1.0
Neutrophils 10^9/l	1.00-1.50	0.75-0.99	0.50-0.74	<0.50
Platelets 10^9/l	75-99	50-74	20-49	<20 or diffuse petechiae
Prothrombin time	1.01-1.25x ULN	1.26-1.50x ULN	1.51-3.00x ULN	>3x ULN
Partial Prothrombin time	1.01-1.66x ULN	1.67-2.33x ULN	2.34-3.00x ULN	>3x ULN
Methaemoglobin	5-9.9%	10.0-14.9%	15.0-19.9%	>20%
BIOCHEMISTRY				
Hyponatraemia mmol/l	130-135	123-129	116-122	<116 or mental status change or seizures
Hypernatraemia	146150	151-157	158-165	>165 or mental status change or seizures
Hypokalaemia mmol/l	3.0-3.4	2.5-2.9 or replacement required	2.0-2.4 or replacement or hospitalisation	<2.0 or paresis or ileus or life-threatening arrhythmia
Hyperkalaemia	5.6-6.0	6.1-6.5	6.6-7.0	>7.0 or life-threatening arrhythmia
Hypocalcaemia mmol/l corrected for albumin	1.99-2.14	1.79-1.98	1.56-1.78	<1.56 or life-threatening arrhythmia
Hypercalcaemia corrected for albumin	2.70-2.93	2.94-3.19	3.20-3.44	>3.44 or life-threatening arrhythmias
Hypomagnesaemia mmol/l	0.60-0.75	0.45-0.59	0.30-0.44	<0.30 or life-threatening arrhythmias
Hypophosphataemia mmol/l	0.64-0.76	0.48-0.63	0.32-0.47	<0.32 or life-threatening arrythmias
Hypoglycaemia mmol/l	3.1-3.6	2.2-3.0	1.7-2.1	<1.7 or mental status change or coma
Hyperglycaemia (fasting)	6.5-9.0	9.1-14.0	14.1-28.0	>28.0 or ketoacidosis or seizures
Bilirubin mmol/l	1.1- 1.5x ULN	1.6- 2.5x ULN	2.6- 5.0x ULN	>5.0x ULN
AST or ALT or GGT U/l	1.26-2.5x ULN	2.6-5.0x ULN	5.1-10x ULN	>10x ULN
Alkaline phosphatase U/l	1.26-2.5x ULN	2.6-5.0x ULN	5.1-10x ULN	>10x ULN
Amylase U/l total or pancreatic or salivary	1.1-1.5x ULN	1.6-2.0x ULN	2.1-5.0x ULN	>5x ULN
Triglycerides (fasting) mmol/l	1.8- 2.2	2.3-5.6	5.7- 10.0	>10.0
Creatinine µmol/l	1.1- 1.5x ULN	1.6- 3.0x ULN	3.1- 6.0x ULN	>6.0x ULN or requires dialysis
Urea mmol/l	1.25-2.5x ULN	2.6- 5.0x ULN	5.1- 10.0x ULN	>10.0x ULN
CK U/l	1.1-2.0x ULN	2.1-4.0x ULN	4.1-6.0x ULN	>6x ULN
URINALYSIS				
Proteinuria	1+ or <0.3% or <3g/l or <1g/day loss	2-3+ or 0.3-1.0% or 3-10g/l or 1-2g/day loss	4+ or >1.0% or >10g/l or 2-3.5g/day loss	nephrotic syndrome or >3.5 g/day loss
Haematuria	microscopic only	gross, no clots	gross + clots	obstruction or requiring transfusion
GASTROINTESTINAL				
Stomatitis/mouth ulcers	mild discomfort, no limits on activity	some limits on eating or talking	eating/ talking very limited	requiring IV fluids
Nausea	mild discomfort, maintains reasonable intake	moderate discomfort, significantly decreased intake	severe discomfort, no significant intake	minimal intake
Vomiting	transient	occasional or moderate	orthostatic hypotension or IV fluids required	shock or hospitalisation required for IV fluids
Diarrhoea	transient or up to 4 loose stools/day	5-7 loose stools/day or nocturnal loose stools	orthostatic hypotension or >7 loose stools/day or requiring IV fluids	shock or hospitalisation required for IV fluids

	Grade 1	Grade 2	Grade 3	Grade 4
Clinical pancreatitis	mild abdominal pain, amylase <2.5x ULN, other causes excluded	moderate abdo. pain, amylase <2.5x ULN, other causes excluded	severe abdo. pain, amylase >2.5x ULN, hospitalised	severe abdo. pain, shock/ hypovolaemia, amylase>5x ULN, hosp.
NEUROLOGICAL				
Consciousness	difficulty in concentration or memory	mild confusion or lethargy <50% waking hours	disoriented or stupor >50% waking hours	coma or seizures
Mood	mild anxiety or depression	treatment required for anxiety or depression	needs assistance due to depression, mania or anxiety	acute psychosis or incapacitated or hospitalised
Headache	mild, no treatment	transient, moderate, requires treatment	severe, responds to first narcotic	intractable needing repeated narcotics
Activities of daily living	mild agitation or difficulty concentrating or confusion	some limitation ADL and minimal treatment required	treatment and assistance needed, severe agitation or confusion	toxic psychosis or hospitalisation
NEUROMUSCULAR				
Muscle strength	subjective weakness	mild objective signs, fully functional	objective weakness, limited function	paralysis
Clinical myopathy	minimal findings	moderate myalgia, may need NSAID, or difficulty climbing stairs or rising from sitting position, able to walk	moderate to severe myalgia needing NSAID, needing assistance walking or for general activities	severe myalgia unrelated to exercise requiring narcotics, unable to walk, or necrosis or oedema
Peripheral neuropathy	mild paraesthesia, numbness, pain or weakness, not treated	moderate paraesthesia, numbness or pain, objective weakness, requires analgesic	severe, narcotic required, interferes with normal activity	intolerable, incapacitating, unable to walk despite narcotics, paralysis
RESPIRATORY				
Bronchospasm	transient, no treatment, >70% peak flow or FEV1	requires treatment, normalises with bronchodilator, 50-70% peak flow or FEV1	no normalisation with bronchodilator, 25-50% peak flow or FEV1	cyanosis or intubated, <25% peak flow or FEV1
OTHER				
Fever, oral, >12 hours	37.7-38.5^{0}C	38.6-39.5^{0}C	39.6-40.5^{0}C	>40.5^{0}C
Fatigue	mild, no decrease in activity	25-50% decrease in activity	>50% decrease in activity, cannot work	unable to care for self
Hypersensitivity	pruritus without rash	localised urticaria	generalised urticaria or angioedema	anaphylaxis
Rash	erythema or pruritus	diffuse, maculopapular rash, dry desquamation	vesiculation, moist desquamation, ulceration	exfoliative dermatitis, mucous membrane involvement, or suspected Stevens-Johnson or erythema multiforme
Haemorrhage	microscopic or occult	mild, no transfusion	gross blood loss, transfused 1-2 units	massive blood loss, transfused >2 units
General	transient, mild, easily tolerated	moderate, discomfort, interrupts usual activity	severe, considerable interference with usual activity	incapacitating or life-threatening

Appendix 7

A. TRIAL CO-INVESTIGATORS

**Brazil**	Dr Paula Gama<br>Clinical Trials Unit - Projeto Praça Onze,<br>Hospital Escola São Francisco de Assis,<br>Universidade Federal do Rio de Janeiro,<br>Av. Presidente Vargas 2863 – 3° Andar,<br>Centro - Rio de Janeiro - RJ - Brazil.<br>CEP: 20210.030	gama@ponze.ufrj.br<br>Tel: +55 21 2273 9073<br>Fax: +55 21 2504-2031
**Italy**	Dr Liviana Della Torre<br>Department of Infectious Diseases<br>San Raffaele Hospital<br>Via Stamira d'Ancona, 20<br>20127 Milan<br>Italy	delletorre.liviana@hsr.it<br>Tel: +39 02 2643 7962<br>Fax: +39 02 2643 7030
**South Africa**	Dr Linda-Gail Bekker<br>Desmond Tutu HIV Centre<br>P O Box 13801<br>Mowbray<br>7705<br><br>Dr Anamika Premrajh<br>HIV Prevention Unit<br>Medical Research Council<br>PO Box 70380<br>Overport<br>4067<br>Durban, Kwazulu-Natal	linda-gail.bekker@hiv-research.org.za<br>Tel: +27 21 650 6959<br>Fax: +27 21 650 6963<br><br>anamika.premrajh@mrc.ac.za<br>Tel: +27 31 2423600<br>Fax: +27 31 2423800
**Uganda**	Dr Heiner Grosskurth<br>MRC Unit on AIDS in Uganda<br>Uganda Virus Research Institute<br>P. O Box 49, Entebbe<br>Uganda<br><br>Dr Anatoli Kamali<br>MRC Unit on AIDS in Uganda<br>Uganda Virus Research Institute<br>P. O Box 49, Entebbe<br>Uganda	heiner.grosskurth@mrcuganda.org<br>Tel: +256 41 320272/320042<br>Fax: +256 41 321137<br><br>anatoli.kamali@mrcuganda.org<br>Tel: +256 41 320272/320042<br>Fax: +256 41 321137
**UK** / **Ireland**	Dr Jonathan Ainsworth<br>Coleridge Unit (T1)<br>North Middlesex Hospital, Sterling Way<br>London N18 1QX<br><br>Dr Jane Anderson<br>Dept Sexual Health<br>Homerton Row<br>London E9 6SR	jonathan.ainsworth@nmh.nhs.uk<br>Tel: +44 20 8887 4185<br>Fax: +44 20 8887 4628<br><br>jane.anderson@qmul.ac.uk<br>Tel: +44 20 8510 5323<br>Fax: +44 20 8510 7978

	Dr Martin Fisher<br>HIV/GUM Research, Elton John Centre,<br>Sussex House, 1, Abbey road,<br>Brighton,<br>BN2 1ES	martin.fisher@bsuh.nhs.uk<br>Tel: +44 1273 664 731<br>Fax: +44 1273 523080
	Dr Elizabeth Hamlyn<br>Clinical Trials Centre,<br>Winston Churchill Wing<br>St. Mary's Hospital, Praed Street<br>London W2 1NY	e.hamlyn@imperial.ac.uk<br>Tel: +44 20 7886 1466<br>Fax: +44 20 7886 6123
	Prof Brian Gazzard<br>Chelsea & Westminster Healthcare NHS Trust<br>Kobler Centre, St. Stephens Centre<br>369 Fulham Road<br>London SW10 9TH	eileen.witney@chelwest.nhs.uk<br>Tel: +44 20 8746 8239<br>Fax: +44 20 8746 5611
	Dr Margaret Johnson<br>Dept. Primary Care & Pop. Sciences<br>Royal Free Hospital, Pond Street<br>London NW3 2QG	sally.allen@rfh.nthames.nhs.uk<br>Tel: +44 20 7830 2775<br>Fax: +44 20 7830 2201
	Dr Fiona Mulcahy<br>Dept of GU Medicine<br>St. James's Hospital, James's Street<br>Dublin 8, Ireland	fmulcahy@stjames.ie<br>Tel: +353 14 537 941<br>Fax: +353 14 544 494
	Dr Chloe Orkin<br>Infection and Immunity<br>St. Bartholomew's hospital<br>West Smithfield,<br>London EC1A 7BE	orkin.chloe@bartsandthelondon.nhs.uk<br>Tel: +44 20 7601 7507<br>Fax: +44 207 601 7341
	Dr Ian Williams<br>Dept. Sexually Transmitted Diseases<br>The Mortimer Market Centre<br>Off Capper Street<br>London WC1E 6AU	iwilliams@gum.ucl.ac.uk<br>Tel: +44 20 7380 9893<br>Fax: +44 20 7380 9778

B. TRIAL COORDINATING CENTRES

**Australia**	Pat Grey<br>Level 9<br>Lowy Packer Building<br>St Vincent's research Precinct<br>405 Liverpool St<br>Darlinghurst, NSW, 2010<br>Australia	pgrey@nchecr.unsw.edu.au<br>Tel: +61 8382 4951<br>Fax: +61 8382 4967
**Kwazulu**	HIV Prevention Unit	ramjeeg@mrc.ac.za

**Natal**	Medical Research Council<br>PO Box 70380<br>Overport<br>4067<br>Durban, Kwazulu-Natal	Tel: +27 31 2423600<br>Fax: +27 31 2423800
**Italy**	Department of Infectious Diseases<br>San Raffaele Hospital<br>Via Stamira d'Ancona, 20<br>20127 Milan<br>Italy	tambussi.giuseppe@hsr.it<br>Tel: +39 02 2643 7934<br>Fax: +39 02 2643 7903

Appendix 8

SAMPLE Long-term storage of samples patient Information

[Each country to use its own translated Patient Information Sheet and Consent Forms according to local regulatory requirements, on local headed paper]

Long-term storage of samples patient Information

The SPARTAC TRIAL
A multi-centre randomised trial of therapeutic intervention at primary HIV infection

Thank you for your valued participation in the SPARTAC trial. Since you joined the trial your doctors have been collecting samples of blood from you. These samples have been used for various tests related to HIV infection. The SPARTAC investigators would like to continue using your extremely valuable samples after the trial ends in 2010, to help us to find out even more about HIV infection. In order for us to do this, we need your permission to continue to use your samples.

What are we asking you to do?

We are asking you to give your permission to continue using and storing the samples you have given as part of the SPARTAC trial until the end of 2020.

You are being asked because you previously agreed to participate in the SPARTAC trial and to have samples stored until the end of the trial. Research using your samples can help to find answers about HIV infection; how to prevent it, and how to treat it. **We would like to use your stored samples, and other information, for such research.**

What will happen if you say yes?

All you need to do is give your written permission (consent) by signing the form attached to this leaflet. Please keep this information sheet to remind you of what you were asked to do.

Your samples will be stored coded, i.e. without any personal identifying details, only your trial number and date of birth. Some of the samples will be stored at the Peter Medawar Centre in Oxford and the rest at Imperial College in London, and they will all be stored until the end of 2020, after which time any remaining samples will be destroyed. Application for use of these specimens by clinical researchers can only be done through the SPARTAC Trial Steering Committee.

If you agree to take part, you will be allowing the continued use and storage of samples that could help research to benefit those affected by HIV in the future.

What will happen if you say no?

You are free to say no – the choice is yours. Your decision will NOT affect the standard or type of care you will receive from the hospital or doctor, now or in the future. If you say no, this does not mean that you cannot remain in the SPARTAC trial; it just means that we will destroy any samples you have given already, at the end of the trial. We may ask you to help us understand why you said no – but you do not have to tell us.

What happens if you change your mind?

You can change your mind at any time by contacting your hospital or clinic directly, you do not need to tell us why.

If you tell us that you have changed your mind, all samples in storage will be destroyed (at the end of the trial) in the way human tissues and blood from hospitals are normally destroyed.

If you change your mind after December 2010 the samples may have already been used. We cannot recall samples or information from researchers once they have been used. If, by then, your samples have already helped create new knowledge, that new information cannot be undiscovered and will contribute to medical understanding. However, we will request the disposal of any of your samples that remain so that these will not be used in any further research.

What are the benefits to you?

If you allow us to continue to use and store your samples, there may be a direct benefit to you but no guarantee can be made. You will NOT receive the results of some tests that are not part of your routine care. It is possible that you may receive no benefit from being in this study. The information we get from this study may help us treat patients with HIV infection better in the future.

What are the risks to you?

There are NO significant health risks to allowing the use of your samples for research purposes in SPARTAC. You will only be allowing us to use samples which have already been collected as part of the trial.

Your hospital, medical team and the Medical Research Council Clinical Trials Unit, who co-ordinate the SPARTAC trial, will take every security precaution to prevent researchers from obtaining any information that identifies who you are. The only people who will know your identity are the hospital staff and a few trained staff dealing with patient records at your hospital/clinic who are bound by a professional duty to protect your privacy. Researchers will NOT be provided with any personal information such as your name, address or phone number. The information that they will be given for their work relates to your disease and treatment only and will NOT be directly linked to your identity. The link to your identity will be securely stored by your hospital/clinic so that if a researcher gets approval to do a follow up study, hospital/clinic may be able to obtain further information about you and your progress. Researchers will, therefore, NOT be able to contact you directly about their research in future.

When samples from the SPARTAC trial are provided to researchers they are obliged to only use the samples for the research they said they would do. Researchers will be bound by a strict agreement to ensure this. Your samples will be used only for medical research and will not be provided for any other purpose.

If you have any questions or concerns about the donation of samples and information or the possible uses of them, please ask the person discussing this with you and seeking your consent.

Long-term storage of samples Consent Form

The SPARTAC TRIAL: A Multi-centre, randomised trial of therapeutic intervention at primary HIV infection (PHI)

Please initial box if you agree

I have read the SPARTAC long-term storage of samples Patient Information sheet (*insert version*)	
I have had an opportunity to ask questions and discuss this information	
I have received satisfactory answers to all of my questions	
I understand that the samples I have already given for the SPARTAC trial will be used and stored until the end of 2020.	
I understand that after the end of 2020 any of my samples that have not been used will be destroyed.	
I accept that I have given my consent voluntarily to the storage of these samples and that I am free to withdraw my consent at any time and the samples will be destroyed.	

____

_Name of patient Date Signature

____

_Name of researcher Date Signature

IMPORTANT: One signed original to be given to patient (with a copy of the Patient Information Sheet)
One signed original to be kept on file by the researcher
One signed photocopy to be kept in the hospital notes
